# Protective effect of cilostazol on vascular injury in rats with acute ischemic stroke complicated with chronic renal failure

**DOI:** 10.1007/s43188-023-00217-w

**Published:** 2023-12-13

**Authors:** Ru Sun, Qun Gu, Xufeng Zhang, Ruiqi Zeng, Dan Chen, Jingjing Yao, Jingjing Min

**Affiliations:** Department of Neurology, the First People’s Hospital of Huzhou, First affiliated Hospital of Huzhou University, Huzhou, China

**Keywords:** Chronic renal failure (CRF), Stroke, Cilostazol, Vascular injury

## Abstract

Chronic renal failure (CRF) resulting in vascular calcification, which does damage to blood vessels and endothelium, is an independent risk factor for stroke. It has been reported that cilostazol has a protective effect on the focal cerebral ischemic infarct. However, its impact on vascular injury in CRF combined stroke and its molecular protection mechanism have not been investigated. In this study, we carried out the effect of cilostazol on CRF combined stroke rats, and the results confirmed that it improved the neurobehavior, renal function as well as pathologic changes in both the kidney and brain. In addition, the inflammation and oxidative stress factors in the kidney and brain were suppressed. Moreover, the rates of brain edema and infarction were decreased. The injured brain-blood barrier (BBB) was recovered with less Evans blue extravasation and more expressions of zonula occludens-1(ZO-1) and occludin. More cerebral blood flow (CBF) in the ipsilateral hemisphere and more expression of CD31 and vascular endothelial growth factor (VEGF) in brain and kidney were found in the cilostazol group. Furthermore, cell apoptosis and cell autophagy became less, on the contrary, proteins of vascular endothelial growth factor receptor 2 (VEGFR2) after the cilostazol treatment were increased. More importantly, this protective effect is related to the pathway of Janus Kinase (JAK)/signal transducer and activator of transcription 3 (STAT3), mammalian target of rapamycin (mTOR), and the hypoxia inducible factor-1α (HIF-1α). In conclusion, our results confirmed that cilostazol exerted a protective effect on the brain and kidney function, specifically in vascular injury, oxidative stress, cell apoptosis, cell autophagy, and inflammation response in CRF combined with stroke rats which were related to the upregulation of JAK/STAT3/mTOR signal pathway.

**Supplementary Information:**

The online version contains supplementary material available at 10.1007/s43188-023-00217-w.

## Introduction

Chronic renal failure (CRF) is a global health concern that is irreversible and can result in cardiovascular disease. Most CRF patients are asymptomatic, and eventually, only experience its typical side effects. Patients who do not need dialysis and typically have glomerular filtration rates > 15 mL/min may receive conservative treatment, whereas others may benefit from alternative therapies (hemodialysis, peritoneal dialysis, and kidney transplantation) [1]. Stroke as a complication of CRF is the third most common cause of death and has high morbidity and disability rates [2]. Collectively, stroke and CRF cause a negative socioeconomic impact and lower quality of life. Few studies have examined how CRF progression affects brain-kidney interactions in stroke pathogenesis, and the global prevalence of the stroke-CRF combination remains unclear [3].

Studies have shown that CRF patients are at a higher risk of stroke than healthy individuals [4]. There is a growing evidence that the combination of CRF and stroke triggers a variety of pathogenic mechanisms such as inflammation, oxidative stress, neurohormone imbalances, uremic toxin formation, and vascular calcification, which worsen the endothelium and blood vessel conditions [5]. CRF patients are more likely to develop cognitive dysfunction, dementia, transitory infarctions, and white matter lesions [6]. The blood–brain barrier (BBB)-crossing uremic toxins induced by chronic renal illness results in cognitive impairment and neurodegeneration [3]. Additionally, CRF causes vascular risk factors that lead to diabetes, atherosclerosis, hypertension, and atrial fibrillation. Additionally, CRF hinders the eligibility of stroke patients for various stroke treatments, accelerates the course of the disease, and decreases recovery outcomes [7].

Cilostazol increases cAMP levels, inhibits phosphodiesterase type III, and has antiplatelet properties; compared to other antiplatelet medications, it has fewer hemorrhagic side effects. It also has several other properties. A study by Kim revealed that cilostazol exerts antidepressant effects after ischemic stroke [8], and the effect of cilostazol and aspirin as a pretreatment against subsequent transient focal cerebral ischemia has been studied in rats [9]. In addition, we noticed that cilostazol disrupts the Interleukin 6 (IL-6)/Janus activated kinase 2 (JAK2)/signal transducer and activator of transcription-3 (STAT3)/suppressor of cytokine signaling (SOCS3) pathway in brain injury in Huntington’s disease, causing the destruction of BBB and increasing the density of blood vessels in rats [10]. Another study showed that cilostazol activates the JAK2/STAT3 pathway to protect mice against myocardial ischemia and reperfusion injury [11]. Cilostazol alleviates brain injury by inhibiting the JAK/STAT3 pathway. However, the direct molecular mechanism by which cilostazol is involved in stroke-CRF combination remains unclear.

Based on these observations, we aimed to explore whether cilostazol reduces vascular injury in rats with CRF combined with stroke and investigate its possible mechanism of action.

## Materials and methods

### Animals and experiments

Forty male Sprague Dawley (SD) rats (6–8 weeks old) obtained from the Zhejiang Weitong Lihua Laboratory Animal Technology Co., Ltd. (animal production license no: SCXK (Zhe) 2019-0001), weighing 180–220 g, were kept under constant temperature (20–24 °C), humidity (55%), 12 h light and dark cycle, and wind change times 15–20 times/h. The animal research was approved by the Ethics Committee of the Animal Center of Zhejiang Eyong Pharmaceutical Research and Development Center (animal use license number: SYXK (Zhe) 2021–0033).

### Establishment of middle cerebral artery occlusion (MCAO) combined CRF model

Forty rats were divided into five groups of eight rats each: control, sham, MCAO + CRF, cilostazol, and aspirin. In the treatment group, cilostazol (30 mg/kg; 73963-72-1, Sigma, China) or aspirin (10 mg/kg; 50-78-2, Sigma, China) was administered daily by gavage for 7 days. In the sham group, the perirenal fat was removed from the kidney, and no nephrectomy was performed. Based on previous studies [12, 13], CRF rats underwent a complete nephrectomy on the right kidney and a two-third nephrectomy on the left kidney, simultaneously. Antibiotics were used to prevent incision infection in each group during the procedure. After the CRF rats were anesthetized, as described in a previous study [14], a nylon thread was prepared, and the external and common carotid arteries were ligated using a silk thread. The prepared nylon cord was then inserted along the common carotid artery into the left internal carotid artery, the vascular clamp was released, the threaded plug was advanced to obstruct the ipsilateral middle cerebral artery, and the threaded plug was fixed above the incision of the left common carotid artery to complete the modeling of MCAO in rats. Neither ligation nor obstruction was performed in the sham group.

### Body weight, urine volume, and 24-h urinary protein level

After 7 days of administration, body weight, urine protein content, and total urine volume in 24 h were measured.

### Modified neurological severity score (mNSS)

The mNSS is frequently used for neurobehavioral evaluation after MCAO. The score range is 0–18; the higher the score, the more severe the neurological impairment is. Each group of rats was trained before testing.

### Sample collection

On the seventh day, the drug was administered for 30 min, and the mNSS score was evaluated. Blood was collected from the submaxillary vein, and brain and kidney tissues were collected and quickly placed on an ice plate to determine the brain water content. The remaining samples were used for molecular and biochemistry experiments, and some were stained in sections.

### Determination of brain water content

The brain tissue was weighed, and after diluting the excess water and blood stains on the surface using disposable sterile gauze, the brain tissue was weighed using a balance, and the weight was noted as wet mass. Subsequently, the brain tissue was incubated in the oven at 60 °C for 48–96 h, and the brain tissue was repeatedly weighed until the mass no longer changed (termed as the dry mass). Brain water content = (wet mass − dry mass)/wet mass × 100%.

### Triphenyltetrazolium chloride (TTC) staining

Brain tissues were coronally sectioned with a thickness of 2 mm. They were stained in 1% TTC at 37 °C for 30 min and fixed in 10% formaldehyde solution for 6 h. The infarct volume was calculated using the Medical Image Processing System software.

### Nissl staining

Nissl staining was performed according to the manufacturer’s instructions. After deparaffinizing and rehydrating coronal slices, the slides were stained for 5 min at 37 °C in Nissl Staining Solution (C0117, Beyotime, Jiangsu, China). The ImageJ software was used to count the cells.

### Hematoxylin–eosin (H&E) staining

The brain tissues were fixed, dehydrated, and immersed in wax. The tissues were cut into 5 μm slices and affixed to the anti-peeling slides. The slices were treated at 60 °C for 1–2 h, dewaxed, hydrated with xylene and gradient ethanol, and stained with H&E staining (G1005, Servicebio, Wuhan, China). Finally, ethanol in increasing concentrations was added for dehydration. After vitrification with xylene, the slices were sealed with neutral balsam and observed under a microscope.

### Quantitative real-time polymerase chain reaction PCR (qRT-PCR)

Pure brain tissue RNA was extracted using TRIzol (B511311; Sangon Biotech, Shanghai, China) and transcribed into cDNA using a reverse transcription kit (CW2569; Jiangsu Cowin Biotech). The primers, diethypyrocarbonate (DEPC), cDNA, and SYBR Green (RR820A; Takara, Beijing, China) were used to prepare the corresponding system for amplification products in the PCR instrument. The primer sequences are listed in Table 1. The fold changes of mRNA were calculated using the 2^−ΔΔCT^ method.

**Table 1:** Primer sequence

Gene	Forward primer (5′–3′)	Reverse primer (5′–3′)
Rat VEGF	TCATCAGCCAGGGAGTCTGT	TTAACTCAAGCTGCCTCGCC
Rat VEGFR2	ACGACTGAAAGCCCAGATTGT	AGCTGAAATCAAGCCCCACG
Rat β-actin	AAGGCCAACCGTGAAAAGAT	GCTCGAAGTCTAGGGCAACA

### TUNEL assay

The TUNEL assay was performed following the manufacturer’s instructions. Deparaffinized tissue slices was treated with Proteinase K (G1205, Servicebio, Wuhan, China) for 15 min in a humid environment, followed by incubation sections in 3% hydrogen peroxide for 10 min and terminal deoxynucleotidyl transferase (G1501, Servicebio, Wuhan, China) labeling buffer at 37 °C for 1 h. TUNEL-positive cells were stained red; nuclei were stained with diamidinylphenyl indole (DAPI) to observe the TUNEL-positive cells.

### Evans blue (EB) staining

One hour before anesthetization with isoflurane, EB physiological saline solution (E8010, Solarbio, Beijing, China) was injected into the femoral vein of the mice to ascertain the EB content. The conjunctiva of the eyes and limbs turned blue after the injection, and after 1 h of circulation, heart perfusion was performed. The brains of the mice were promptly sectioned, collected, and placed under an inverted fluorescence microscope with blue excitation light to observe EB leakage. The amount of EB present in the brain tissue was determined using a fluorescence spectrophotometer.

### Cerebral blood flow (CBF) evaluation

At 7 days post-treatment, the dynamic blood flow value of the wound was measured using a laser Doppler blood flow meter (SKK-1100, Shenzhen Reward Life Technology Co., LTD, China). Before testing, the skin was excised to expose the skull, and a MSP200XP surface probe was placed on the wound surface of the brain. Three points were chosen on each wound surface; each point was recorded for 30 s, and the PowerLab Chart5 v5.2.2 image analysis software was used for analysis. Color-coded images represented different perfusion levels. The average value of the measurements from these three points was considered as the blood flow value of the intraoral wound.

### Enzyme-linked immunosorbent assay (ELISA) assay

The serum levels of endothelin-1 (ET-1) (MM-0560R1; MEIMIAN, Jiangsu, China), nitrous oxide (NO) (MM-20607R1; MEIMIAN, Jiangsu, China), interleukin-1β (IL-1β) (MM-0047R1; MEIMIAN, Jiangsu, China), IL-6 (MM-0190R1; MEIMIAN, Jiangsu, China), tumor necrosis factor-α (TNF-α) (MM-0180R1; MEIMIAN, Jiangsu, China), endothelial nitric oxide synthase (eNOS) (RX300651R; RUIXING, Fujian, China), malonaldehyde (MDA) (S0131S; Biyuntian Biotechnology Co., LTD. Jiangsu, China), glutathione (GSH) (S0053; Biyuntian Biotechnology Co., LTD. Jiangsu, China), and superoxide dismutase (SOD) (S0101S; Shanghai Biyuntian Biotechnology Co., LTD. China) were tested using the ELISA kits following the manufacturer’s instructions. The serum creatinine (Scr) and blood urea nitrogen (BUN) levels of cilostazol in MCAO rats were determined using an automatic biochemical analyzer.

### Immunofluorescence assay

Brain tissues were fixed and then stabilized in 0.5% Triton X-100. After blocking with blocking buffer, the tissues were incubated with occludin (DF7504, Affinity, Jiangsu, China), LC3 (AF5402, Affinity, Jiangsu, China), and zonula occludens-1 (ZO-1) (21773-1-AP, proteintech, Shanghai, China) overnight at 4 °C. The tissues were then incubated with an anti-rabbit antibody and counterstained with DAPI. The cells were observed under a microscope.

### Immunohistochemistry (IHC) assay

The brain tissue sections were dewaxed with xylene, followed by addition of ethanol in decreasing concentrations for tissue rehydration, and the addition of antigen repair solution. Subsequently, the sections were washed with hydrogen peroxide to block endogenous peroxidase, sealed with bovine serum, and incubated overnight at 4 °C with transforming growth CD31 (AF6191, affinity, Jiangsu, China), vascular endothelial growth factor (VEGF) (ab72807, abcam, Shanghai, China), and Caspase 3 (Ab184787, abcam, Shanghai, China). On the next day, the sections were incubated in HRP secondary antibodies, and DAB (G1212, Servicebio, Wuhan, China) was added. The positive expression of DAB was brown-yellow, and the nuclei were stained with hematoxylin. Finally, ethanol in increasing concentrations was added for dehydration, and vitrification was performed with xylene. The slices were sealed with neutral balsam and observed under a microscope.

### Western blotting

Pure protein was extracted from the brain tissue, and the protein concentration was measured using the bicinchoninic acid (BCA) method. After adding the loading buffer, the protein was denatured via boiling. The total protein was separated by electrophoresis, and the corresponding proteins were transferred to a polyvinylidene fluoride membrane (PVDF) membrane. The non-specific antigen was blocked with 5% milk, and the proteins on the membrane were incubated with the target antibodies listed in Table 2. After incubation at 4 °C overnight, the proteins were incubated with secondary antibodies. The images were captured using an ECL chemiluminescence imager.

**Table 2:** Antibody information

Reagent	Company	No.	Dilution ratio	Lot
Bcl-2 antibody	Affinity	AF6139	1:1000	11o9905
Bax antibody	Affinity	AF0120	1:1000	44q6915
Caspase-3 antibody	Affinity	AF6311	1:1000	33d5960
LC3 antibody	Affinity	AF5402	1:1000	35y4418
Beclin1 antibody	Affinity	AF5128	1:1000	86s3201
p62 antibody	Affinity	AF5384	1:1000	43z8686
VEGF antibody	Affinity	DF7470	1:1000	71j8125
VEGFR2 antibody	Affinity	AF6281	1:1000	82z6076
JAK1 antibody	Affinity	AF5012	1:1000	95r2924
p-JAK1 antibody	Affinity	AF2012	1:1000	34u0236
STAT3 antibody	Affinity	AF6294	1:1000	15x8824
p-STAT3 antibody	Affinity	AF3293	1:1000	74m1478
HIF-1α antibody	Affinity	AF1009	1:1000	60h4847
mTOR antibody	Affinity	AF6308	1:1000	64m3376
p-mTOR antibody	Affinity	AF3308	1:1000	63a3839
Anti-rabbit IgG, HRP-linked antibody	CST	7074	1:6000	29
β-actin antibody	Affinity	AF7018	1:10,000	12w2944

## Results

### Cilostazol attenuated inflammation and oxidative stress response and exert protective effect in the kidney of rats with MCAO combined with CRF

**Fig. 1:**
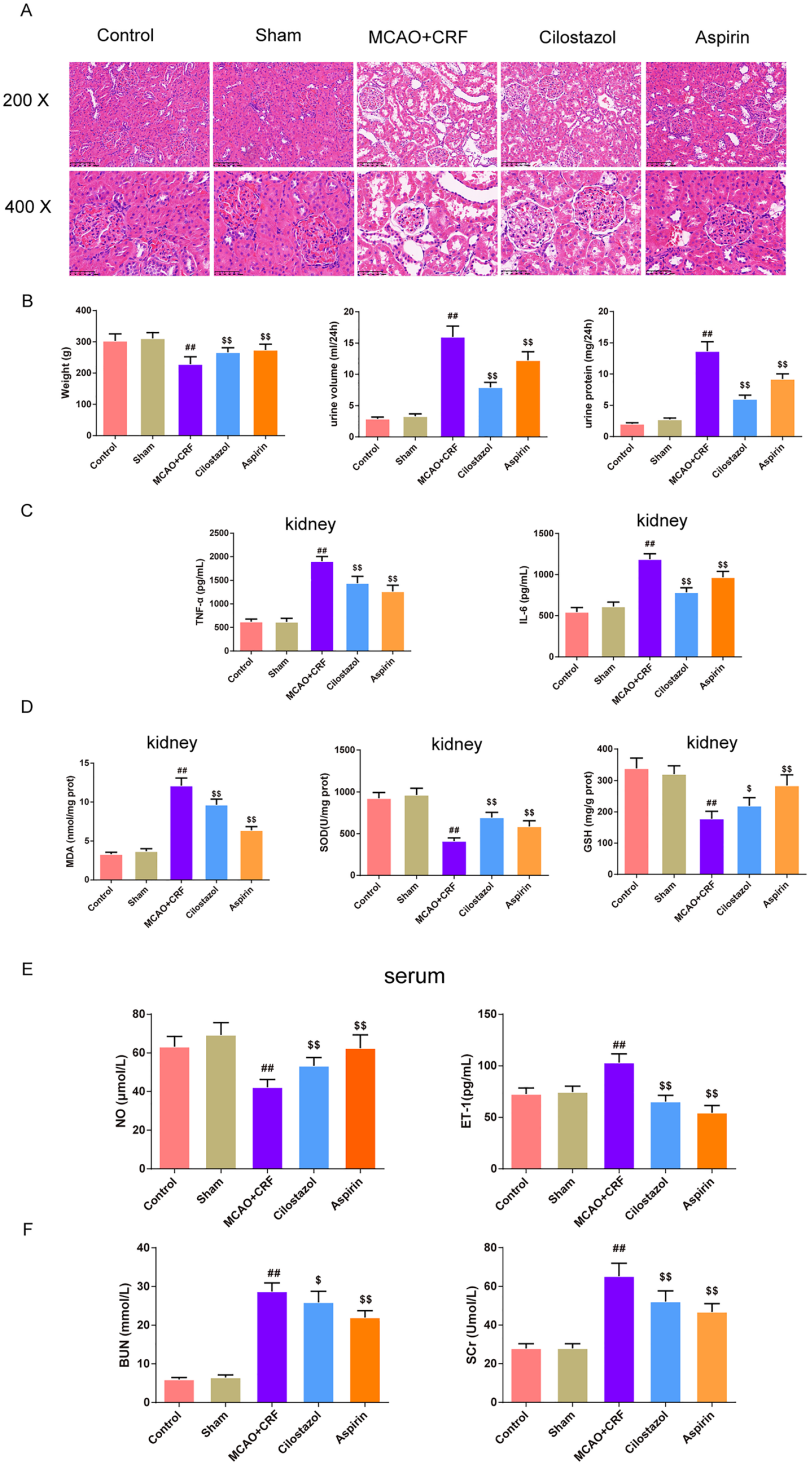
Cilostazol attenuated inflammation and oxidative stress response and exert protective effect in the kidney of rats with MCAO combined with CRF. **a** The histomorphology of in kidney in rats was observed by HE staining (magnification × 200, scale bar: 100 μm; magnification × 400, scale bar: 50 μm), *n* = 3; **b** The weight and the urine volume and urine protein were record in each group,* n* = 8; The expression of inflammatory factors TNF-α, IL-6 in kidney (**c**), the oxidative stress factor MDA, SOD, GSH in kidney (**d**) and NO, ET-1, BUN and Scr in serum were tested by the ELISA kits,* n* = 8. ^##^*P* < 0.01 versus sham group. ^$^*P* < 0.05, ^$$^*P* < 0.01 versus MCAO + CRF group. (*Note*: HE: hematoxylin–eosin, TNF-α: tumor necrosis factor-α, IL-6: interleukin-6, MDA: malonaldehyde, SOD: superoxide dismutase, GSH: glutathione, NO: nitrous oxide, ET-1: endothelin-1, BUN: blood urea nitrogen, Scr: serum creatinine, ELISA: enzyme-linked immunosorbent assay, MCAO + CRF: middle cerebral artery occlusion + chronic renal failure rats)

As shown in Fig. 1a, the kidney tissue structures of the control and sham groups were normal with clear layers, and no obvious kidney injury was observed. In the MCAO + CRF group, the renal tissue was severely damaged, with atrophy of the glomeruli, enlargement of the tubule cyst lumen, and atrophy of the lumen and lumen epithelium at different sizes. Compared to the MCAO + CRF group, cilostazol and aspirin groups showed improved tissue structure. Treatment with cilostazol and aspirin promoted the recovery of body weight, urine volume, and urine protein levels that were abnormally stimulated in the MCAO + CRF group (Fig. 1b, *P* < 0.01). In addition, inflammatory factors in the kidney, such as TNF-α and IL-6, were increased in the MCAO + CRF group compared to in the sham group; they were suppressed in the cilostazol and aspirin groups (Fig. 1c, *P* < 0.01). MDA expression was elevated in the kidneys of MCAO + CRF rats compared to in the sham rats, and treatment with cilostazol and aspirin promoted the recovery of MDA levels (Fig. 1d, *P* < 0.01). The expression of SOD and GSH was reduced in the MCAO + CRF group compared to in the sham group; however, it was enhanced in the cilostazol and aspirin groups (Fig. 1d, *P* < 0.01). In addition, in the serum, the expression of NO was reduced, but ET-1, BUN, and Scr levels were increased in the MCAO + CRF group compared to in the sham group (Fig. 1e, f, *P* < 0.01). This effect was reversed by cilostazol and aspirin treatment (Fig. 1e, f, *P* < 0.01).

### Cilostazol alleviated brain injury and exert a neuroprotective effect in rats with MCAO combined with CRF

**Fig. 2:**
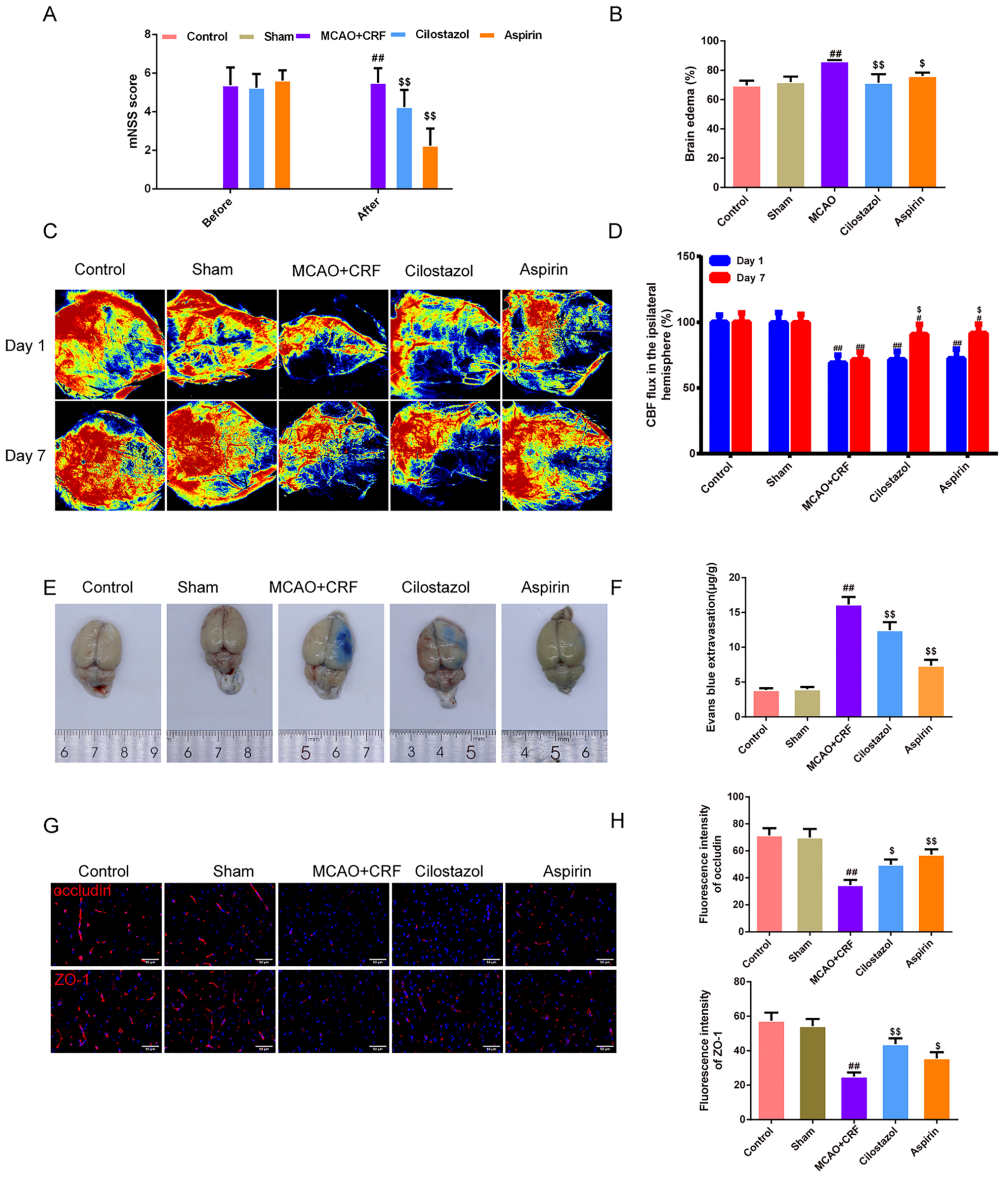
Cilostazol alleviated brain injury and exert a neuroprotective effect in rats with MCAO combined with CRF. **a** mNSS score was used to evaluate the severe of the neurofunction in each group of rats, *n* = 8; **b** The percentage of water content in brain was used to assess the brain edema in each group of rats, *n* = 3; **c**, **d** The dynamic blood flow value of the wound in each group of rats after modeling and 7 days post-treatment was measured using a laser doppler blood flow meter, *n* = 3; **e**, **f** Evans Blue staining and the indicator of extravasation was used to assess the permeability of the BBB,* n* = 3; **g**, **h** Immunofluorescence was used to stain occluding and ZO-1 in rats’ brain respectively and the fluorescence intensity of them were recorded in each group (magnification, × 400, scale bar: 50 μm), *n* = 3; ^##^*P* < 0.01 versus sham group. ^$^*P* < 0.05, ^$$^*P* < 0.01 versus MCAO + CRF group. (*Note*: mNSS: Modified Neurological Severity Score; ZO-1: zonula occludens-1, MCAO + CRF: middle cerebral artery occlusion + chronic renal failure rats)

**Fig. 3:**
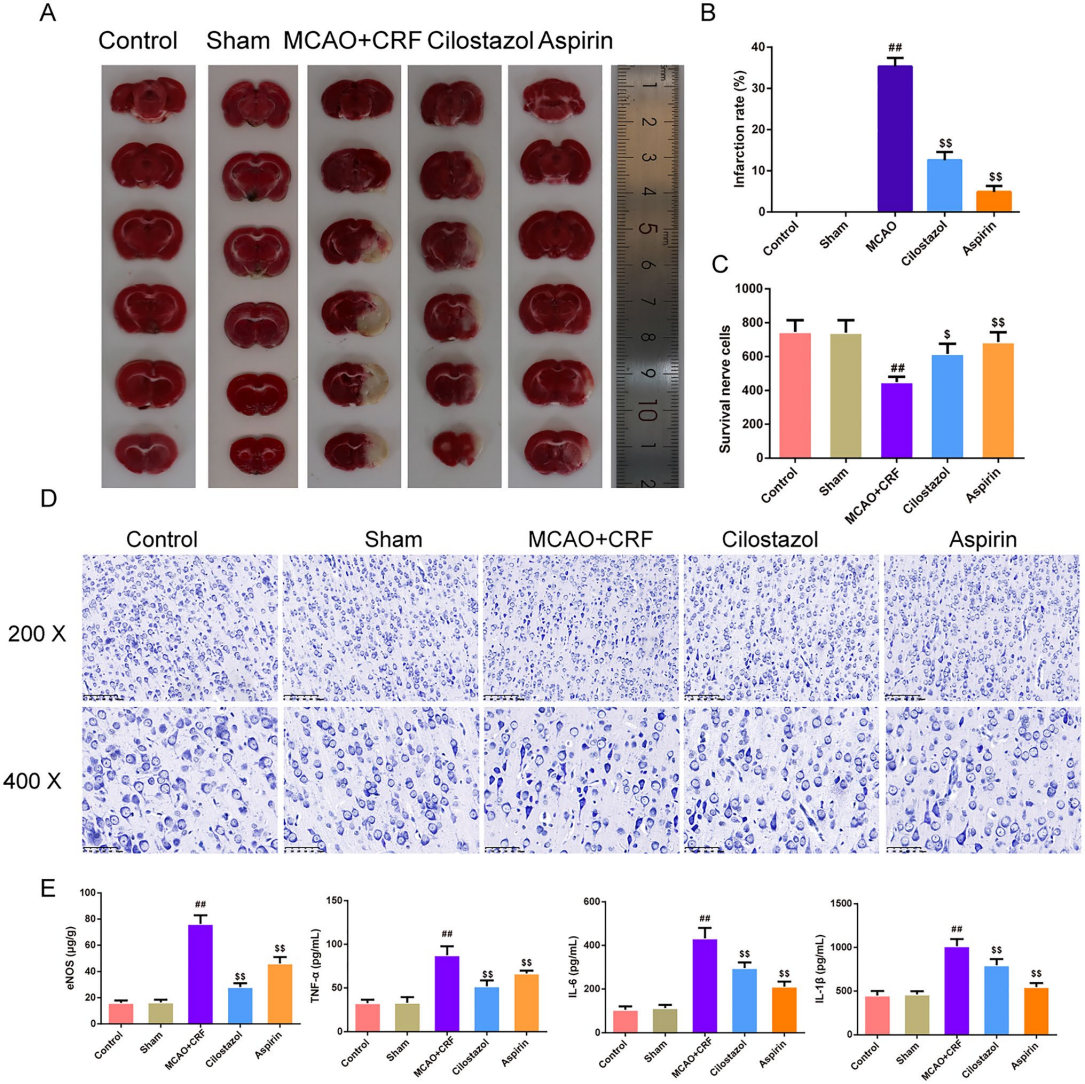
Cilostazol alleviated brain injury and exert a neuroprotective effect in rats with MCAO combined with CRF. **a**, **b** The TTC staining was applied to calculate the infarction rate in rats,* n* = 3. **c**, **d** The histomorphology of in brain cortex in rats was observed by Nissl staining (magnification × 200, scale bar: 100 μm; magnification × 400, scale bar: 50 μm), and the survival nerve cells in each group were calculated,* n* = 3. **e** The content of eNOS, TNF-α, IL-6, IL-1β in brain were tested by ELISA kit,* n* = 8. ^##^*P* < 0.01 versus sham group. ^$^*P* < 0.05, ^$$^*P* < 0.01 versus MCAO + CRF group. (*Note*: TTC: triphenyltetrazolium chloride; BBB: blood brain barrier; eNOS: endothelial nitric oxide synthase, MCAO + CRF: middle cerebral artery occlusion + chronic renal failure rats)

As shown in Fig. 2a, the mNSS score was lower in the cilostazol and aspirin groups than in the MCAO + CRF group (*P* < 0.01). In addition, rats in the MCAO + CRF groups had more severe brain edema than those in the sham group; however, this effect was reversed by cilostazol and aspirin treatment (Fig. 2b, *P* < 0.01). As shown in Fig. 2c, d, the dynamic blood flow value of the wound in each group 7 days post-treatment was measured using a laser Doppler blood flow meter; the results showed that on day 1, the CBF in the ipsilateral hemisphere of the rat brain in the MCAO + CRF, cilostazol, and aspirin groups was lower than that in the sham group (*P* < 0.01). On day 7, CBF in the ipsilateral hemisphere of the rat brain in the MCAO + CRF group was lower than that in the sham group (*P* < 0.05 or *P* < 0.01), whereas the cilostazol and aspirin groups had higher CBF in the ipsilateral hemisphere of the brain than that in the MCAO + CRF group (*P* < 0.05). Subsequently, we used Evans blue staining and immunofluorescence to test the permeability of the BBB and found that brain tissues of MCAO + CRF rats had more extravasation and fewer components of the BBB, including lower expression of occludin and ZO-1, than those of brain tissues of the sham rats (Fig. 2e, f, *P* < 0.01). In contrast, cilostazol and aspirin groups had reduced Evans blue extravasation and enhanced levels of occludin and ZO-1 compared to those in rats of the MCAO + CRF group (Fig. 2g, h, *P* < 0.05 or *P* < 0.01). As for the infarction rate (Fig. 3a, b), we used TTC staining to observe the brain infarction area, and the results showed that MCAO + CRF rats had the highest brain infarction rate among all groups, and the infarction was suppressed by cilostazol and aspirin treatment (*P* < 0.01). In addition, MCAO + CRF rats had more neuronal injury, as demonstrated by the Nissl staining, compared to that of the sham rats (Fig. 3c, d, *P* < 0.01). Cilostazol and aspirin groups had a higher survival of nerve cells than that observed in rats of the MCAO + CRF group (Fig. 3c, d, *P* < 0.01). Furthermore, we used the ELISA kits to test for pro-inflammatory factors (Fig. 3e). The results revealed that the expression levels of eNOS, TNF-α, IL-6, and IL-1β were higher in MCAO + CRF rats than in sham rats. Cilostazol and aspirin groups showed a decrease in the above expressions compared to the MCAO + CRF group (*P* < 0.01).

### Cilostazol reduced cell apoptosis and autophagy and improved angiogenesis in the brain tissues of rats with MCAO combined with CRF

**Fig. 4:**
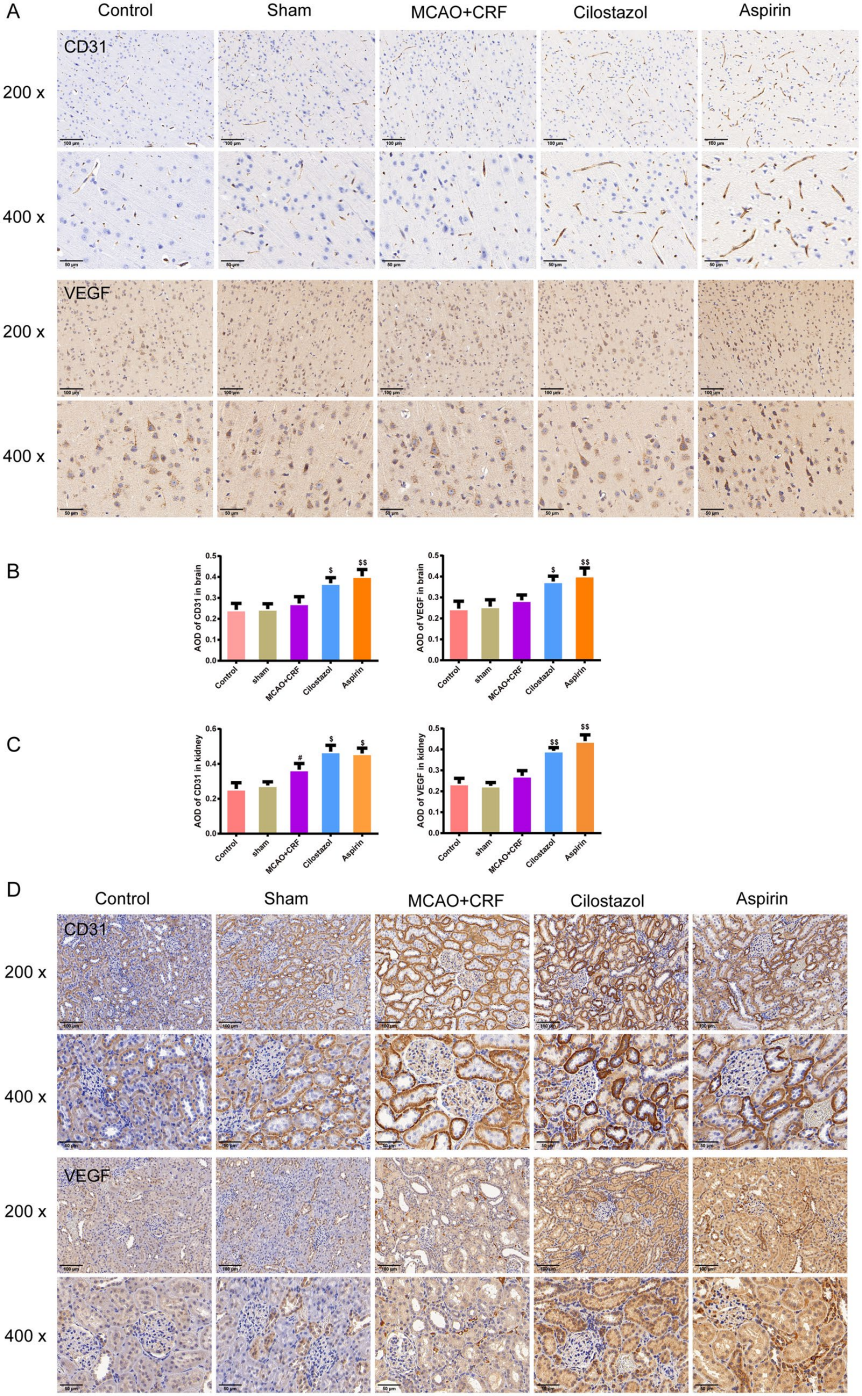
Cilostazol improved angiogenesis in the brain tissues of rats with MCAO combined with CRF. **a**, **b** The positive expression of the CD31 and VEGF in brain was stained by immunohistochemistry, and the average of the density of the CD31 and VEGF was represented as graphs (magnification × 200, scale bar: 100 μm; magnification × 400, scale bar: 50 μm),* n* = 3. **c**, **d** The positive expression of the CD31 and VEGF in kidney was stained by immunohistochemistry, and the average of the density of the CD31 and VEGF was represented as graphs (magnification × 200, scale bar: 100 μm; magnification × 400, scale bar: 50 μm),* n* = 3. ^#^*P* < 0.05 versus sham group. ^$^*P* < 0.05, ^$$^*P* < 0.01 versus MCAO + CRF group. (*Note*: VEGF: vascular endothelial growth factor; MCAO + CRF: middle cerebral artery occlusion + chronic renal failure rats).

**Fig. 5:**
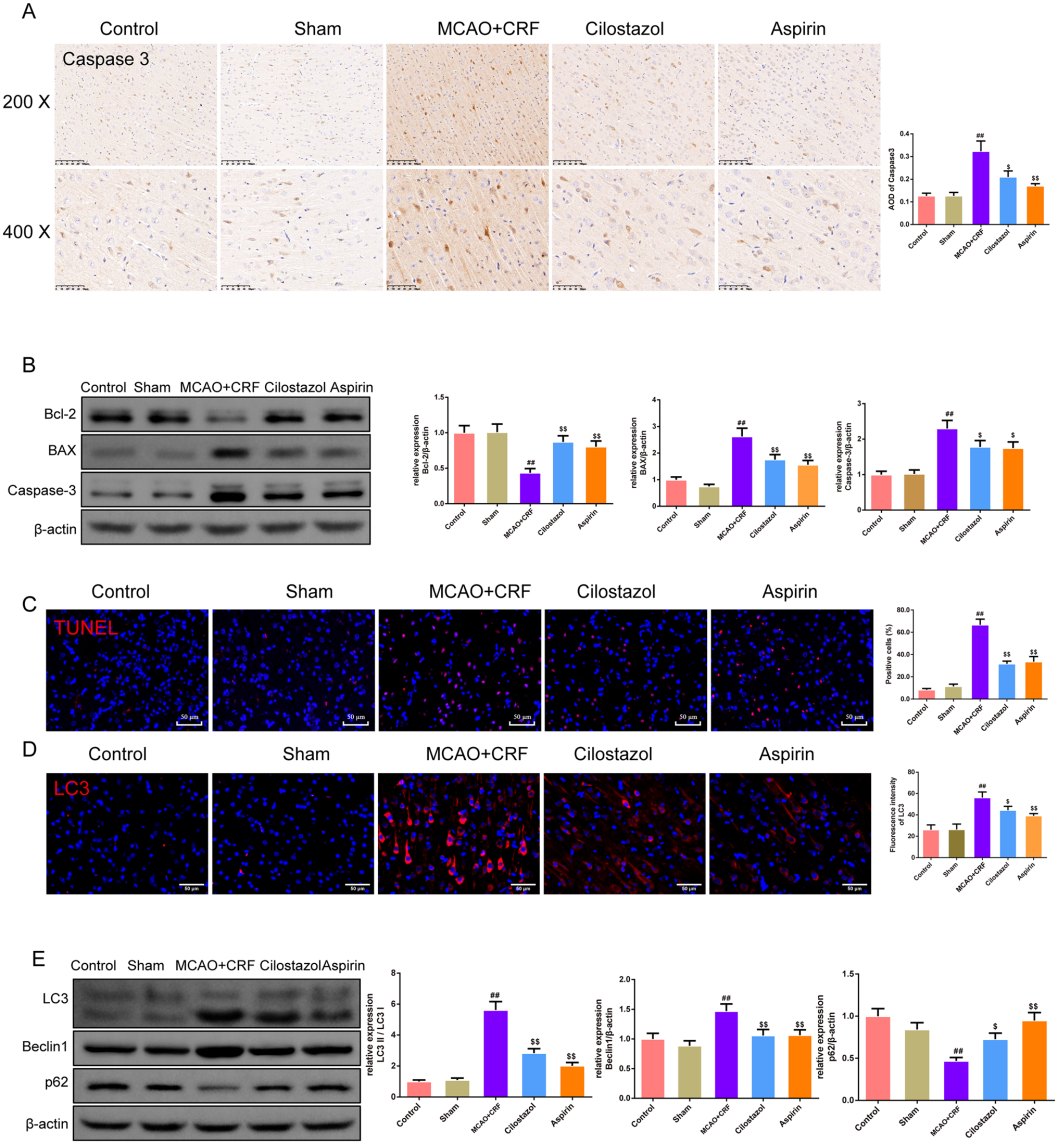
Cilostazol reduced cell apoptosis and autophagy in the brain tissues of rats with MCAO combined with CRF. **a** The positive expressions of Caspase 3 of the brain tissues in rats were examined by immunohistochemistry (magnification × 200, scale bar: 100 μm; magnification × 400, scale bar: 50 μm),* n* = 3; **b** Western bolt was used to evaluate the expression of the Bcl-2, BAX and Caspase 3 protein in brain of rats in each group,* n* = 3; **c** The percentage of the TUNEL-positive cells in the brain tissues was recorded by TUNEL staining,* n* = 3; **d** Immunofluorescence was used to stain LC3 in brain tissues of rats in each group,* n* = 3; **e** Western bolt was used to evaluate the expression of the LC3, Beclin 1 and p62 proteins in brain of rats in each group,* n* = 3; ^##^*P* < 0.01 versus sham group. ^$^*P* < 0.05, ^$$^*P* < 0.01 versus MCAO + CRF group. (*Note*: Bcl-2: B-cell lymphoma-2, BAX: Bcl-2-associated X; MCAO + CRF: middle cerebral artery occlusion + chronic renal failure rats)

Immunohistochemical staining was used to measure CD31, VEGF, and Caspase 3 proteins expression, and we found that cilostazol- and aspirin-treated rats had higher CD31 and VEGF expressions in the brain and kidney than those of MCAO + CRF rats (Fig. 4a, b, *P* < 0.05 and *P* < 0.01, respectively). Furthermore, the expression of caspase 3 was higher in the brain of the MCAO + CRF rats than in the sham rats; this expression level was reversed by cilostazol and aspirin treatment (Fig. 5a, *P* < 0.05 and *P* < 0.01, respectively). In addition, the western blotting results confirmed that the MCAO + CRF group had higher expression of caspase 3 and BAX but lower expression of Bcl-2 than those of the sham group, and cilostazol and aspirin treatment caused opposite effects (Fig. 5b, *P* < 0.05 and *P* < 0.01, respectively). As shown in Fig. 5c, the percentage of TUNEL-positive cells was higher in the MCAO + CRF group than in the sham group; this percentage was reduced after cilostazol and aspirin treatment (*P* < 0.01). The results of the LC3 immunofluorescence staining of the brain were comparable among the groups (Fig. 5d, *P* < 0.05 and *P* < 0.01). In addition, western blotting showed that the expression of LC3 and Beclin1 increased, whereas p62 expression decreased in the MCAO + CRF group than in the sham group. This effect was reversed by cilostazol and aspirin treatment (Fig. 5e; *P* < 0.05 and *P* < 0.01, respectively).

### Cilostazol prompted the JAK/STAT3/mTOR pathway in the brain tissues of rats with MCAO combined with CRF

**Fig. 6:**
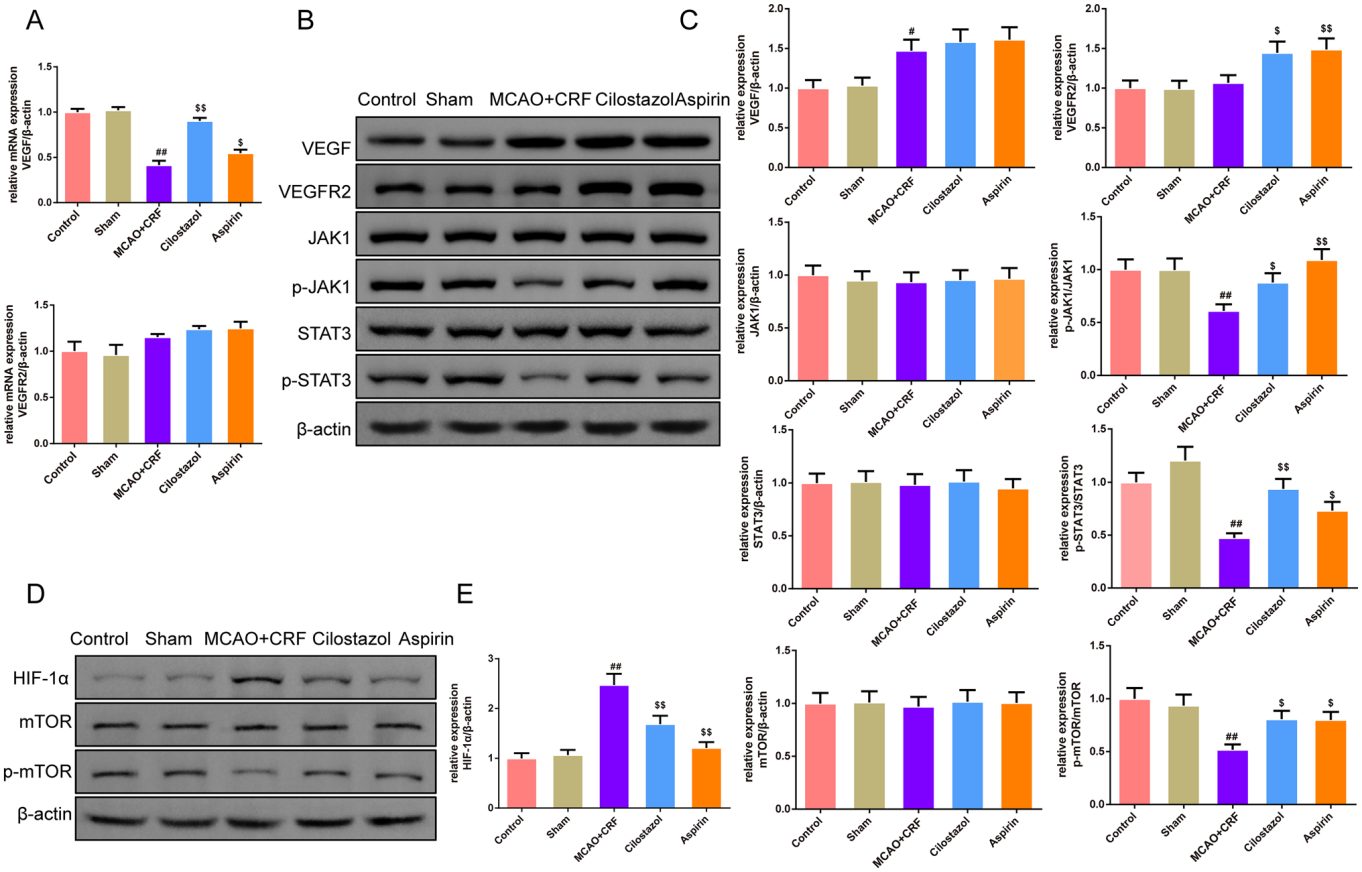
Cilostazol prompted the JAK/STAT3/mTOR pathway in the brain tissues of rats with MCAO combined with CRF. **a** qRT-PCR was used to test the level of the VEGF and VEFGR2 in brain tissues,* n* = 3; **b**–**e** Western bolt was used to evaluate the expression of the VEGF, VEFGR2, JAK1, p-JAK1, STAT3, p-STAT3, HIF-1α, mTOR, p-mTOR in brain of rats in each group,* n* = 3; ^##^*P* < 0.01 versus sham group. ^$^*P* < 0.05, ^$$^*P* < 0.01 versus MCAO + CRF group. (*Note*: qRT-PCR: quantitative real-time PCR; VEGF: vascular endothelial growth factor; VEFGR2: vascular endothelial growth factor receptor 2; MCAO + CRF: middle cerebral artery occlusion + chronic renal failure rats, JAK: Janus Kinase; STAT3: signal transducer and activator of transcription 3; mTOR: mammalian target of rapamycin; HIF-1α: hypoxia inducible factor-1α)

As shown in Fig. 6a, qRT-PCR was used to test the expression of VEGF and vascular endothelial growth factor receptor 2 (VEGFR2), and the results showed that VEGF decreased in the MCAO + CRF group and was enhanced in the cilostazol and aspirin groups (*P* < 0.05 and *P* < 0.01, respectively). However, VEGFR2 expression were not different among the groups (Fig. 6a). As shown in Fig. 6b, e, the expression of VEGFR2 in cilostazol and aspirin groups was higher than that in the MCAO + CRF group (*P* < 0.05 and *P* < 0.01, respectively). In addition, the p-JAK, p-STAT3, and p-mTOR expression in the MCAO + CRF group was lower than that in the sham group; this expression was improved by cilostazol and aspirin treatment (*P* < 0.05 and *P* < 0.01, respectively). The expression levels of JAK, STAT3, and mTOR did not differ among the groups. Additionally, the expression of the hypoxia inducible factor-1α (HIF-1α) was intensified in the MCAO + CRF group compared to the sham group; this expression was reduced by cilostazol and aspirin treatment (both *P* < 0.01).

## Discussion

CRF is rather common (10–13% of the population), irreversible, progressive, and linked to an increased risk of cardiovascular disease [11]. According to the Atherosclerosis Risk in Communities Research, a substantial stroke risk is found in CRF patients. Cilostazol is a potentially effective treatment for intimal hyperplasia occurring after endothelial damage and CRF [15]. In this study, we investigated the effect and mechanism of action of cilostazol in rats with CRF combined with stroke.

First, we successfully established a CRF combined with stroke model in rats, which was reflected in the renal pathological changes observed in the H&E staining. Meanwhile, the 24 h urine protein content, urine volume, body weight, and BUN and Scr serum levels dramatically changed in the CRF model. These results indicated that nephrectomy induced kidney injury in CRF rats. In addition, cilostazol, to some extent, alleviated renal pathological changes and acted as an indicator of renal injury. Specifically, the 24 h urine protein content, urine volume, and BUN and Scr serum levels were restrained after treatment with cilostazol. Simultaneously, aspirin was used as a positive control. Consistently, a study by Lee confirmed that cilostazol ameliorates albuminuria and restores serum albumin levels in rats with type 1 diabetes [16].

Oxidative stress and chronic inflammation are known to contribute to the CRF development, which has been linked to oxidative stress markers such as SOD, oxidized low-density lipoprotein (LDL), homocysteine, and GSH [17, 18]. In our study, the expression of MDA, SOD, and GSH in the kidney and NO and ET-1 in the serum were altered in the CRF model. Similarly, elevated blood levels of pro-inflammatory molecules such as IL-6, TNF-α, osteocalcin, and fibroblast growth factor have been reported in CRF patients [19]. As expected, cilostazol treatment ameliorated CRF-induced inflammation and oxidative stress in the kidneys and serum. A study has verified that cilostazol restrained amikacin-induced nephrotoxicity in rats by reducing the levels of oxidation parameters, including MDA, GSH, SOD, and a significant reduction of inflammatory mediators such as TNF-α, IL-6 expression in the kidney tissue [20].

A slight decline in renal function is associated with some degree of peripheral and central nervous system (CNS) complication [21–24]. Therefore, we established an MCAO model based on CRF rats to simulate stroke complications. The success of the model was confirmed by neurobehavioral scores, CBF, cerebral water content, TTC, and Nissl staining, which indicated that the brain was infarcted and impaired, accompanied with the destruction of BBB after MCAO surgery in CRF rats. The BBB is compromised in the early stages of CRF owing to oxidative stress and low-grade inflammation, which also encourages the infiltration of white blood cells and admission of uremic toxins into the CNS [23]. By reducing endothelial inflammation and apoptotic death, cilostazol pretreatment protects against cold hepatic ischemia–reperfusion injury [25]. In our study, oxidative stress, inflammatory cytokines, and BBB-related junction proteins in brain tissues were significantly altered in the MCAO + CRF group. EB staining showed that BBB integrity was damaged; however, the condition was improved after cilostazol intervention. Cilostazol protects brain tissue cells from apoptosis and autophagy. A previous study using primary rat brain capillary endothelial cells, and the protective effect of cilostazol on the barrier activities of BBB-related endothelial cells [26] indicated that cilostazol exerted a protective effect against stroke after CRF injury.

Cilostazol stimulates angiogenesis in a rat model of myocardial ischemia–reperfusion injury by increasing the number of new blood vessels and VEGF expression [27]. In our study, the expression of VEGF and VEGFR2 was enhanced after cilostazol treatment. According to previous studies, JAK and STAT3 are sufficient to shield the myocardium from apoptosis [28]. The JAK/STAT3 pathway is a crucial signaling pathway involved in several physiological processes, including apoptosis and inflammation [29]. Additionally, we discovered that cilostazol suppressed HIF-1α while greatly increasing the phosphorylation of JAK/STAT3/mTOR proteins. Therefore, our findings reveal a novel signaling pathway involving JAK/STAT3/mTOR, in which cilostazol protects against kidney and brain injuries. However, because our animal model had both brain and kidney injuries, we should further explore more phases of the disease, i.e., the acute and recovery phases, to have an overall observation.

In summary, cilostazol exerted a protective effect on the brain and kidney function in rats with MCAO combined with CRF, specifically against vascular injury, oxidative stress, cell apoptosis, cell autophagy, and inflammatory response. This effect may be related to the upregulation of JAK/STAT3/mTOR pathway. This study provides a basis for further clinical and experimental studies on cilostazol treatment for CRF combined with stroke.

### Supplementary Information

Below is the link to the electronic supplementary material.

Supplementary file 1 (XLSX 68 kb)

Supplementary file 2 (DOCX 257 kb)

Supplementary file 3 (PDF 7883 kb)

## Data Availability

The datasets generated during and/or analyzed during the current study are available from the corresponding author on reasonable request.
