# Supplementary material for: Protective effect of cilostazol on vascular injury in rats with acute ischemic stroke complicated with chronic renal failure
Source: Toxicol Res. 2023 Dec 13;40(2):189–202. doi: 10.1007/s43188-023-00217-w (PMC10959867; doi:10.1007/s43188-023-00217-w)
Supplement: Supplementary file 2 — (DOCX 257 kb) [file 43188_2023_217_MOESM2_ESM.docx]

Comprehensive records of the MCAO surgical procedure, including body weight, body temperature, cerebral blood flow (both pre and post occlusion), and clinical signs are maintained.
