# Supplementary material for: Protective effect of cilostazol on vascular injury in rats with acute ischemic stroke complicated with chronic renal failure
Source: Toxicol Res. 2023 Dec 13;40(2):189–202. doi: 10.1007/s43188-023-00217-w (PMC10959867; doi:10.1007/s43188-023-00217-w)
Supplement: Supplementary file 3 — (PDF 7883 kb) [file 43188_2023_217_MOESM3_ESM.pdf]

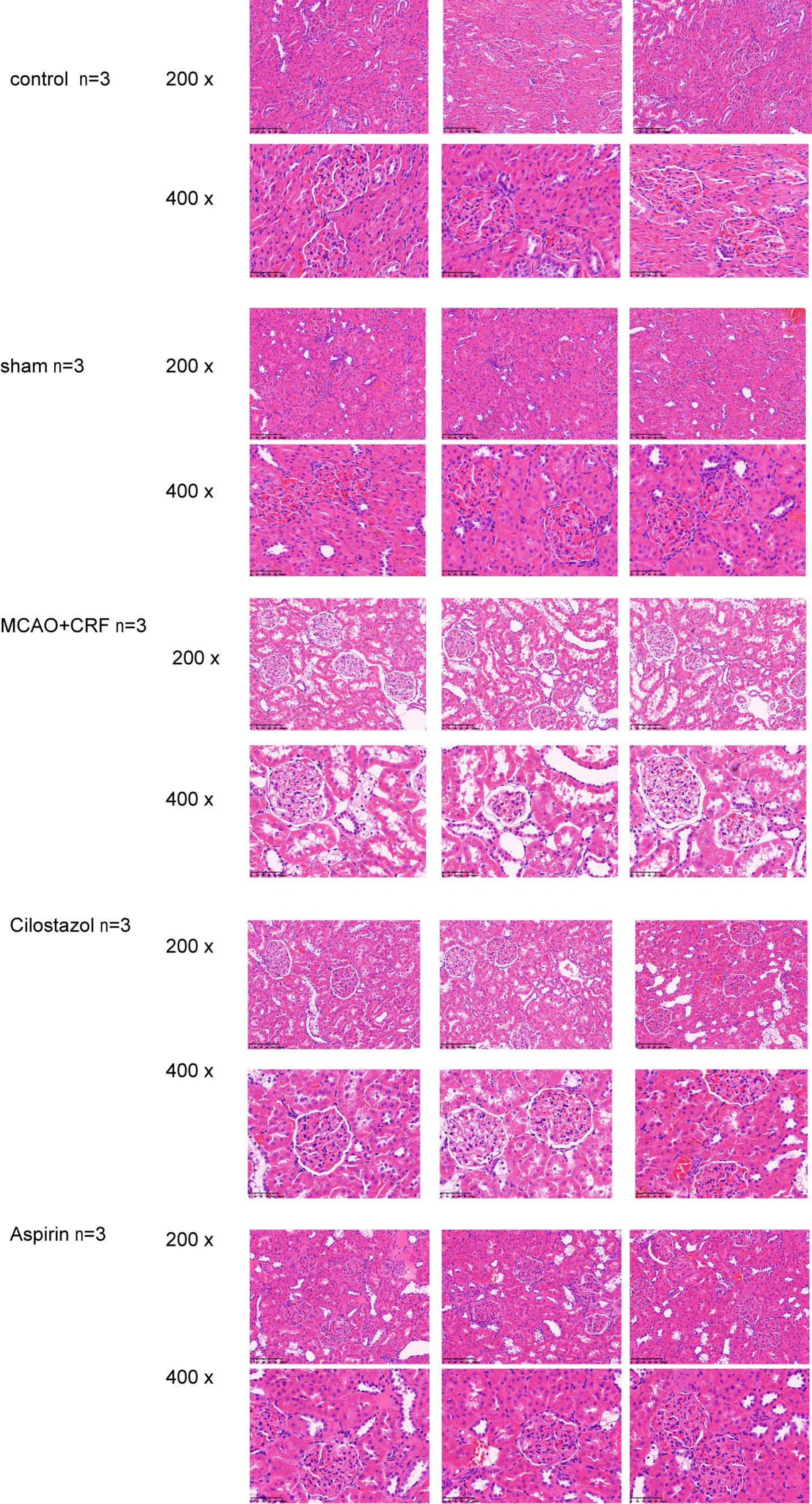
control n=3
200 x
400 x
sham n=3
200 x
400 x
MCAO+CRF n=3
200 x
400 x
Cilostazol n=3
200 x
400 x
Aspirin n=3
200 x
400 x

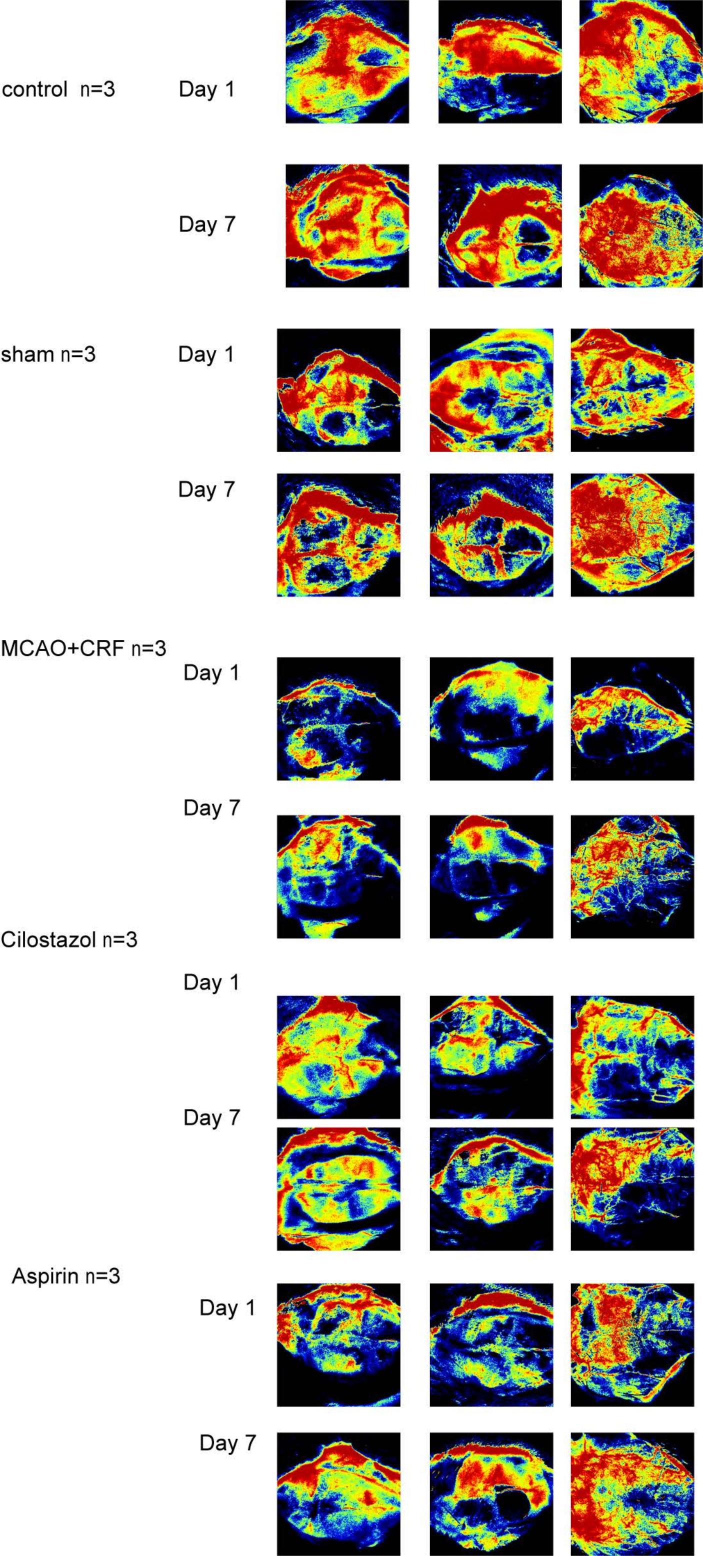
control n=3
Day 1
Day 7
sham n=3
Day 1
Day 7
MCAO+CRF n=3
Day 1
Day 7
Cilostazol n=3
Day 1
Day 7
Aspirin n=3
Day 1
Day 7

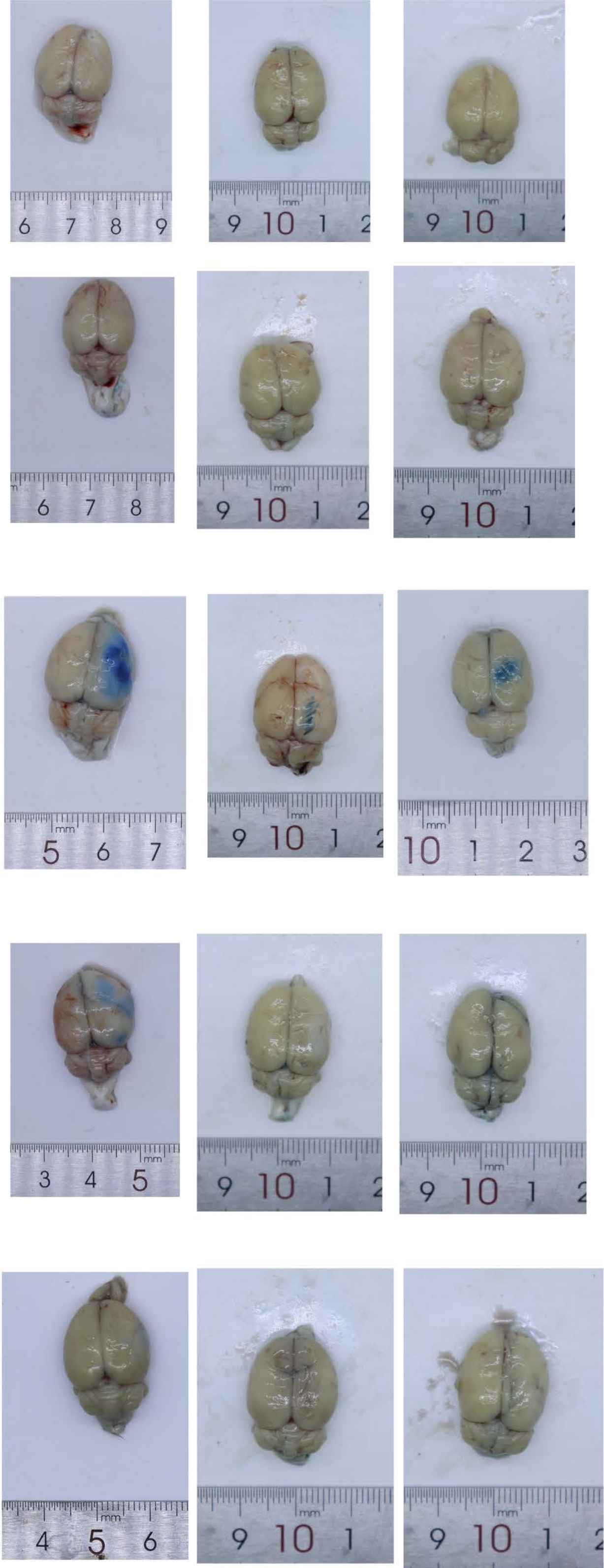

control n=3

sham n=3

MCAO+CRF n=3

Cilostazol n=3

Aspirin n=3

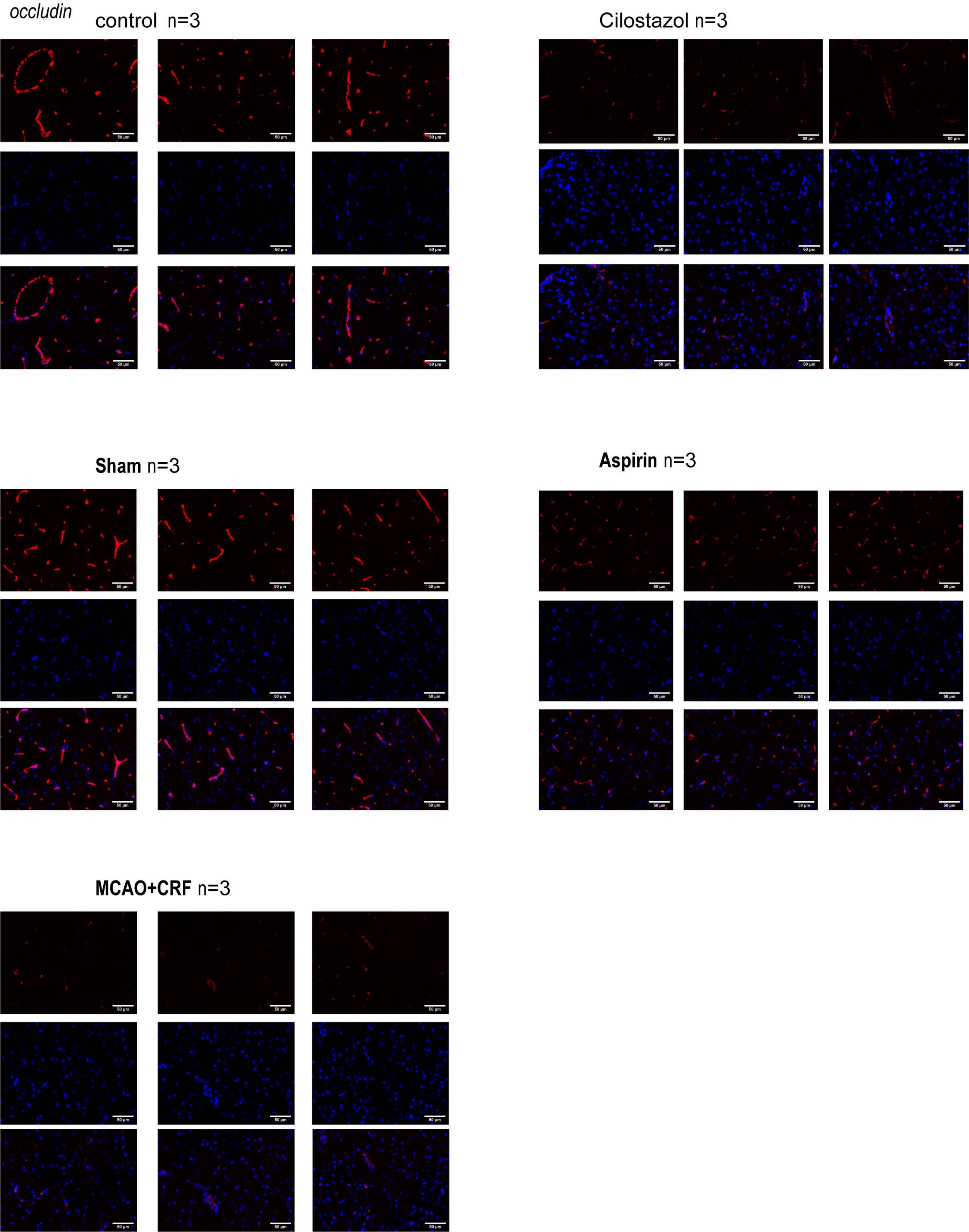
occludin
control n=3
Cilostazol n=3
Sham n=3
Aspirin n=3
MCAO+CRF n=3

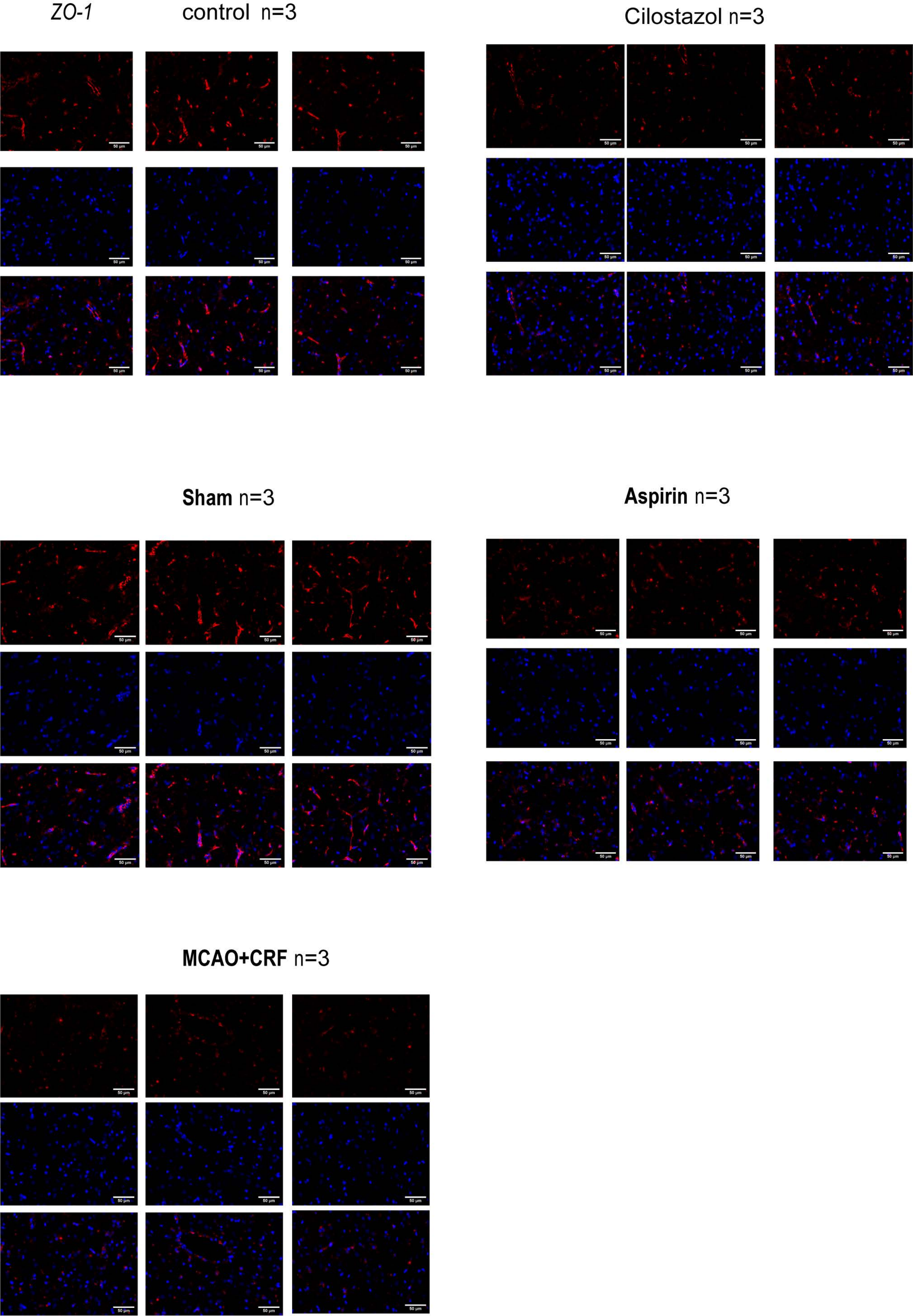
ZO-1
control n=3
Cilostazol n=3
Sham n=3
Aspirin n=3
MCAO+CRF n=3
50 μm

control n=3

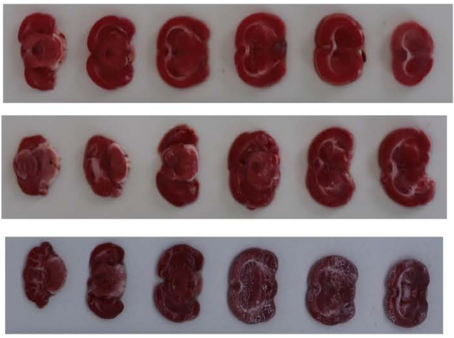

sham n=3

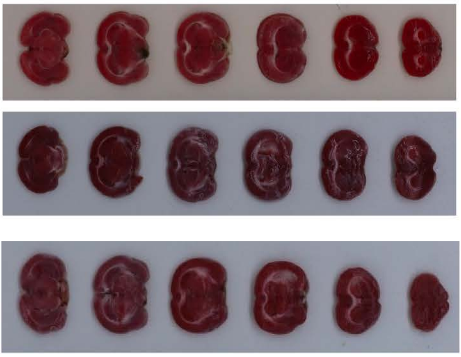

MCAO+CRF n=3

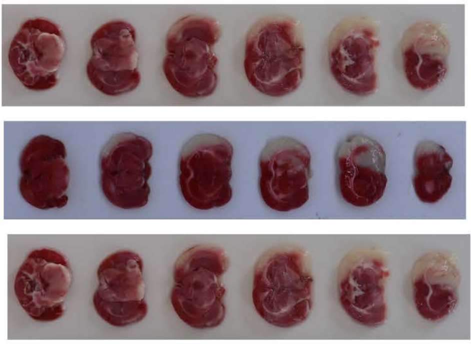

Cilostazol n=3

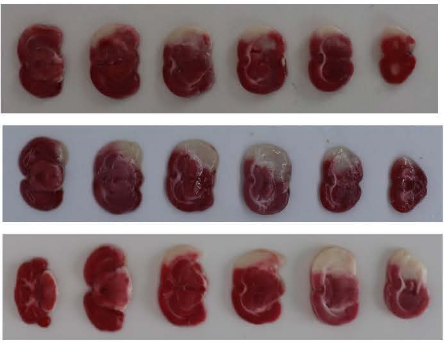

Aspirin n=3

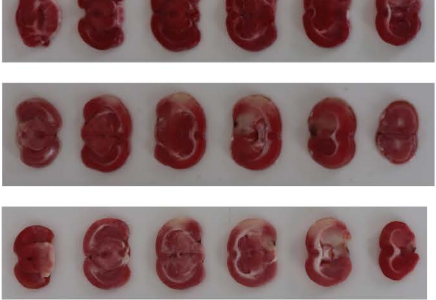

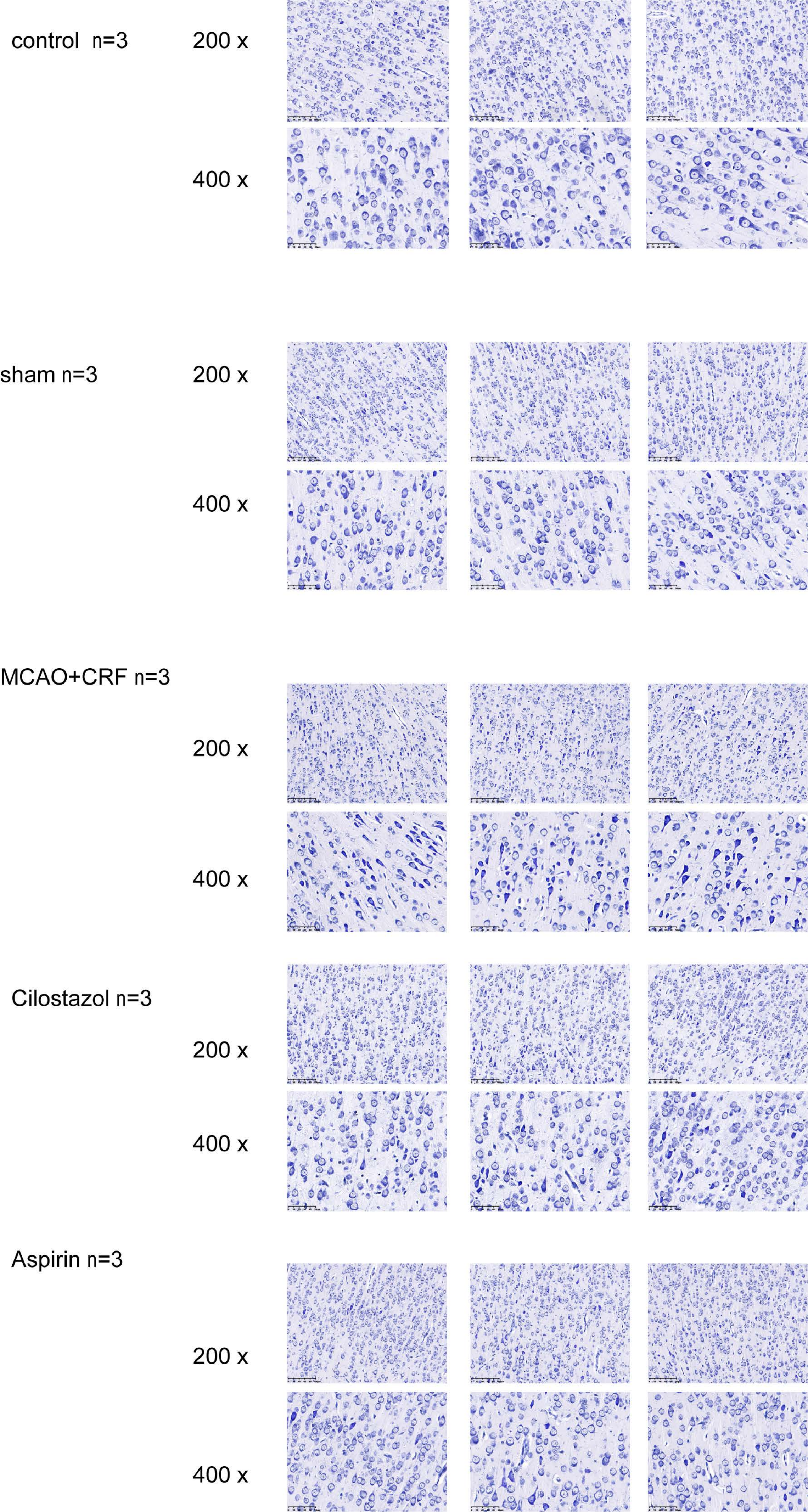
control n=3
200 x
400 x
sham n=3
200 x
400 x
MCAO+CRF n=3
200 x
400 x
Cilostazol n=3
200 x
400 x
Aspirin n=3
200 x
400 x

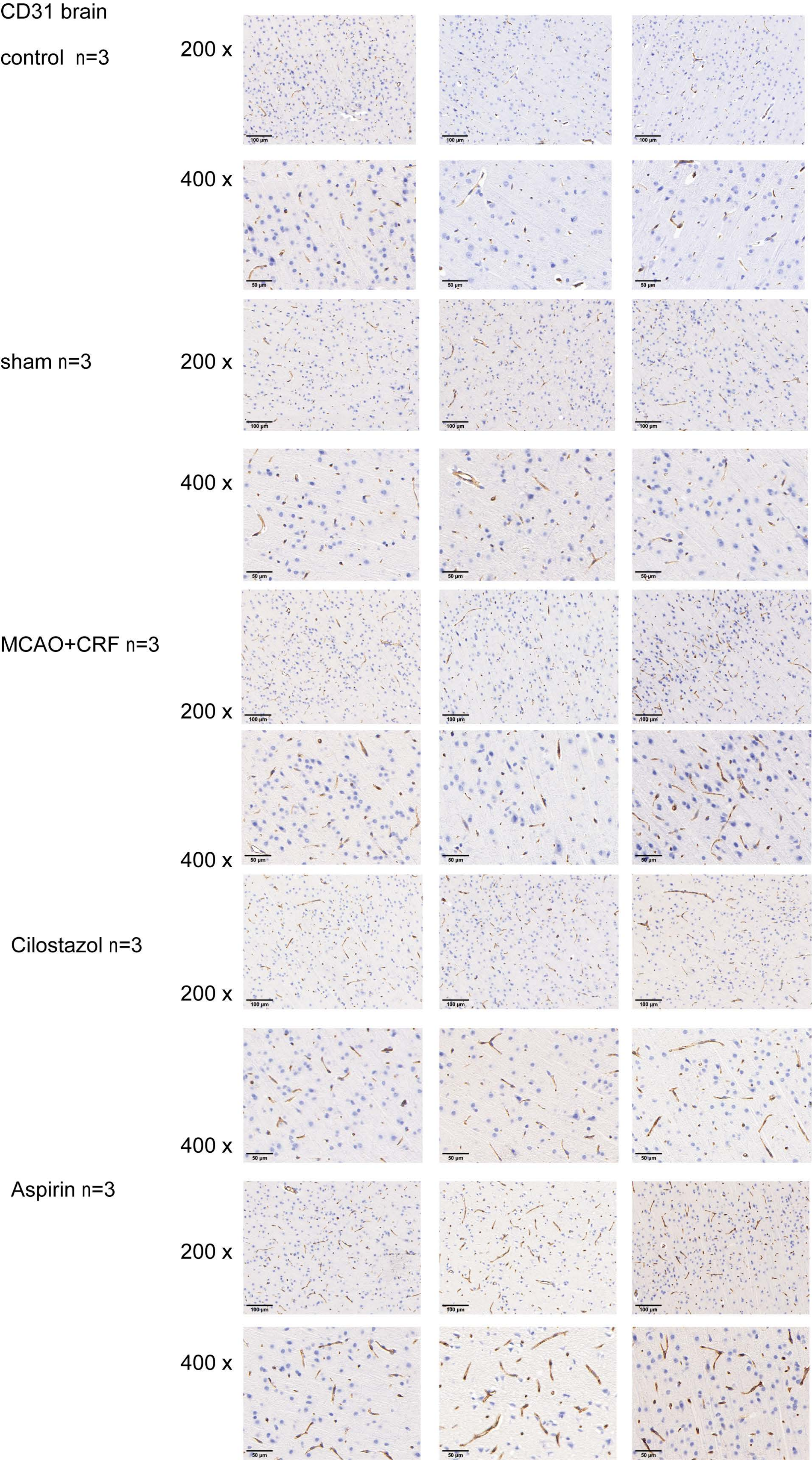
CD31 brain
control n=3
200 x
400 x
sham n=3
200 x
400 x
MCAO+CRF n=3
200 x
400 x
Cilostazol n=3
200 x
400 x
Aspirin n=3
200 x
400 x
100 μm
50 μm

VEGF brain

control n=3 200 x

400 x

sham n=3 200 x

400 x

MCAO+CRF n=3

200 x

400 x

Cilostazol n=3

200 x

400 x

Aspirin n=3

200 x

400 x

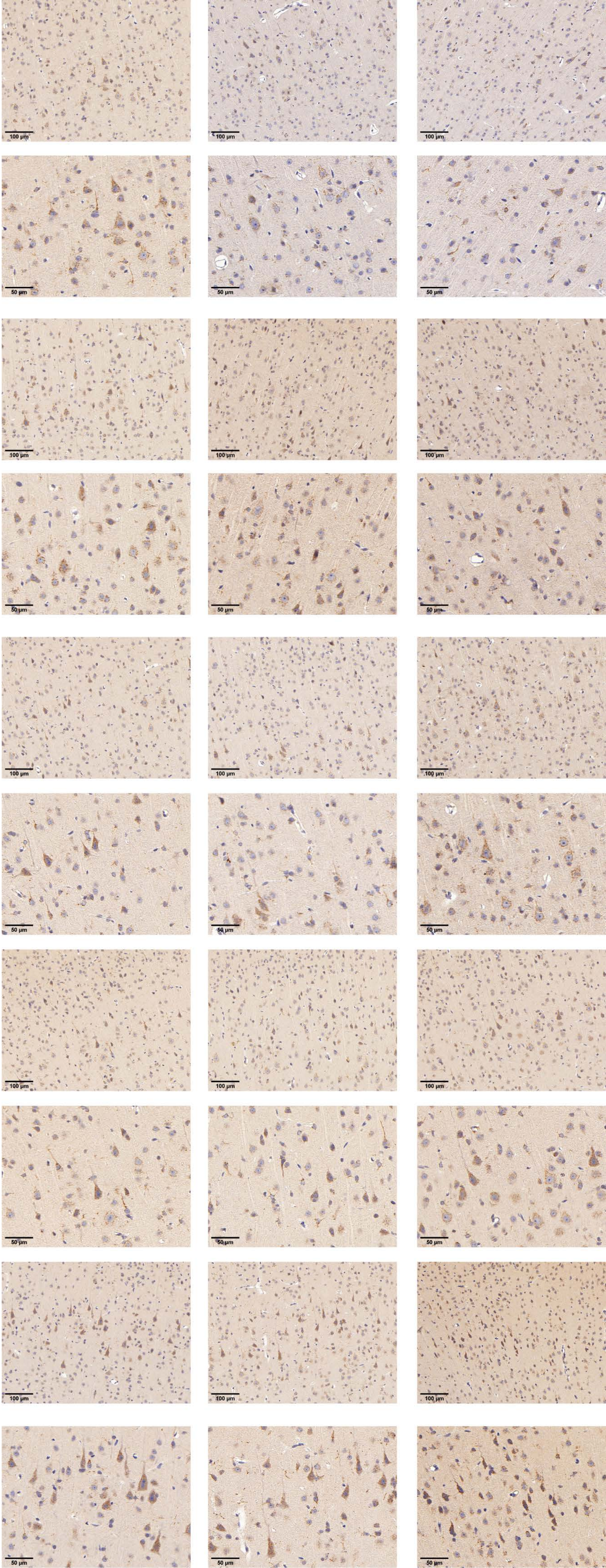

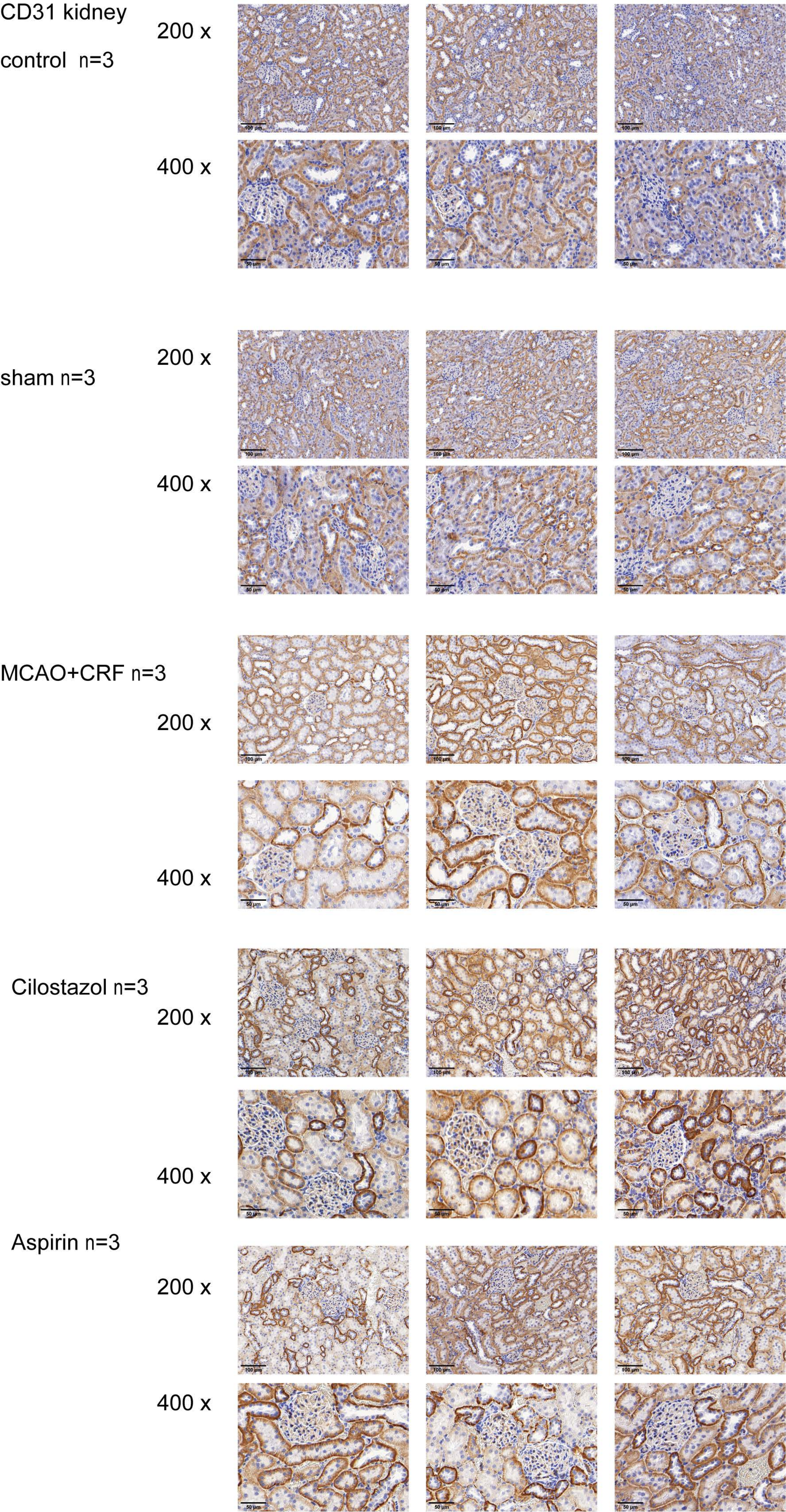

CD31 kidney
control n=3
200 x
400 x
sham n=3
200 x
400 x
MCAO+CRF n=3
200 x
400 x
Cilostazol n=3
200 x
400 x
Aspirin n=3
200 x
400 x
100 μm
50 μm

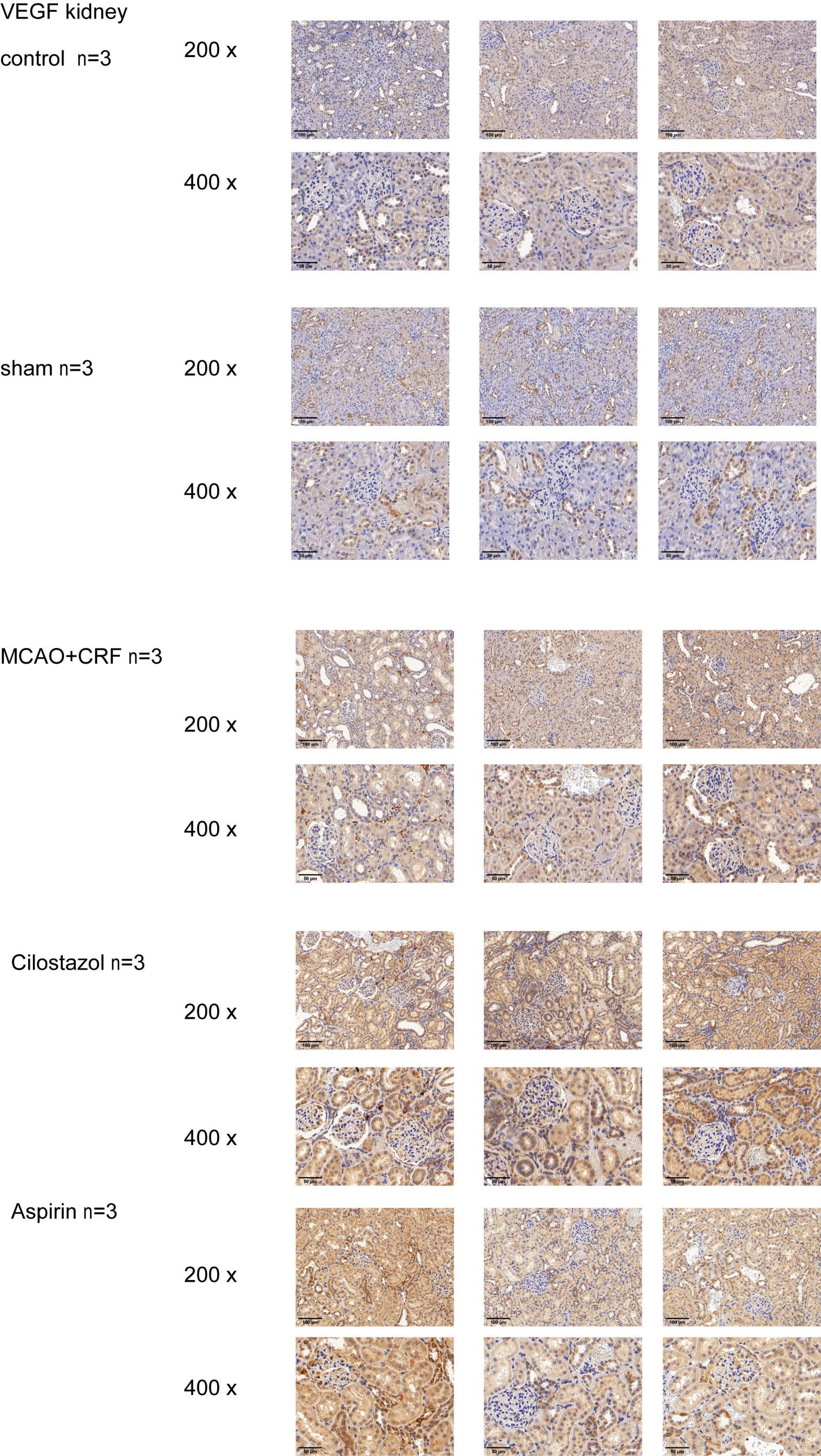

VEGF kidney
control n=3
200 x
400 x
sham n=3
200 x
400 x
MCAO+CRF n=3
200 x
400 x
Cilostazol n=3
200 x
400 x
Aspirin n=3
200 x
400 x

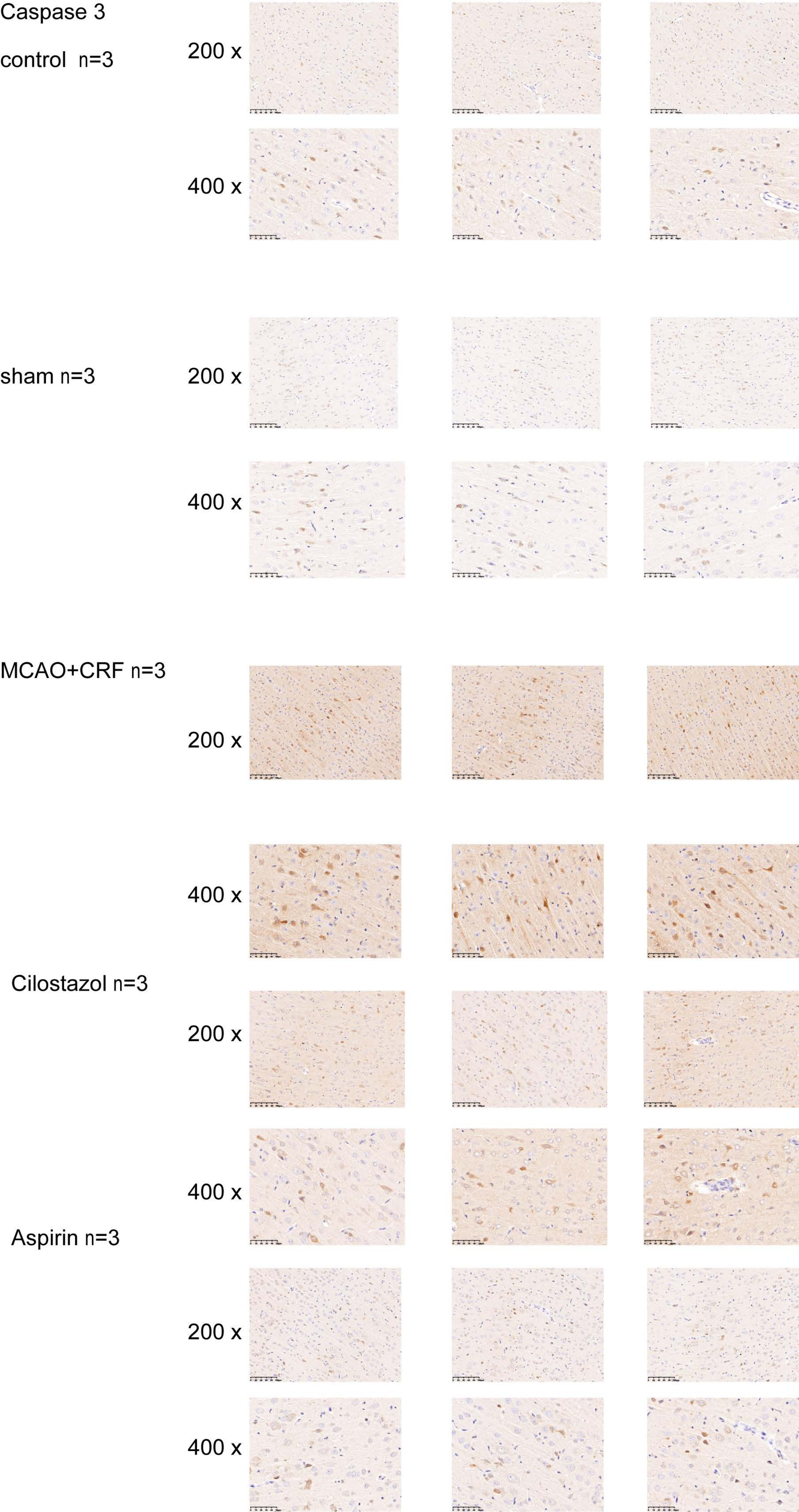
Caspase 3
control n=3
200 x
400 x
sham n=3
200 x
400 x
MCAO+CRF n=3
200 x
400 x
Cilostazol n=3
200 x
400 x
Aspirin n=3
200 x
400 x

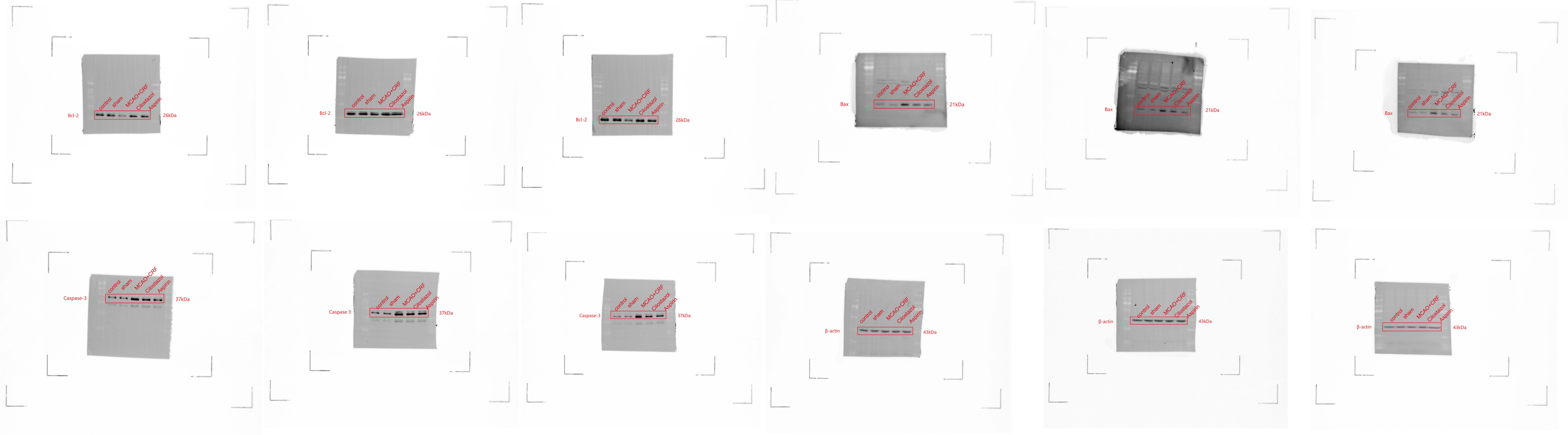
Bcl-2
control
sham
MCAO+CRF
Cilostazol
Aspirin
26kDa
Bcl-2
control
sham
MCAO+CRF
Cilostazol
Aspirin
26kDa
Bcl-2
control
sham
MCAO+CRF
Cilostazol
Aspirin
26kDa
Bax
control
sham
MCAO+CRF
Cilostazol
Aspirin
21kDa
Bax
control
sham
MCAO+CRF
Cilostazol
Aspirin
21kDa
Bax
control
sham
MCAO+CRF
Cilostazol
Aspirin
21kDa
Caspase-3
control
sham
MCAO+CRF
Cilostazol
Aspirin
37kDa
Caspase 3
control
sham
MCAO+CRF
Cilostazol
Aspirin
37kDa
Caspase-3
control
sham
MCAO+CRF
Cilostazol
Aspirin
37kDa
β-actin
control
sham
MCAO+CRF
Cilostazol
Aspirin
43kDa
β-actin
control
sham
MCAO+CRF
Cilostazol
Aspirin
43kDa
β-actin
control
sham
MCAO+CRF
Cilostazol
Aspirin
43kDa

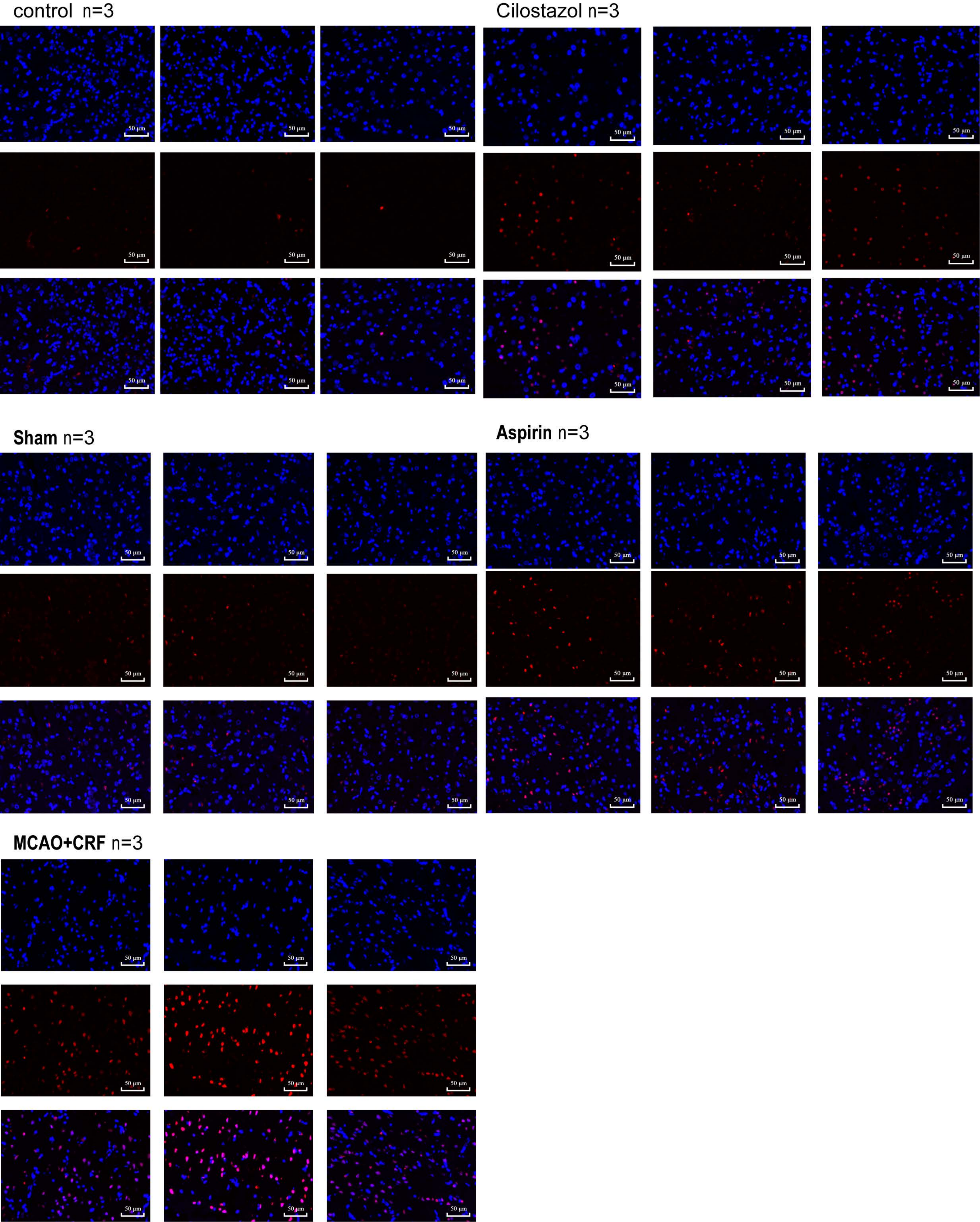

control n=3
Cilostazol n=3
Sham n=3
Aspirin n=3
MCAO+CRF n=3
50 μm

LC3

control n=3

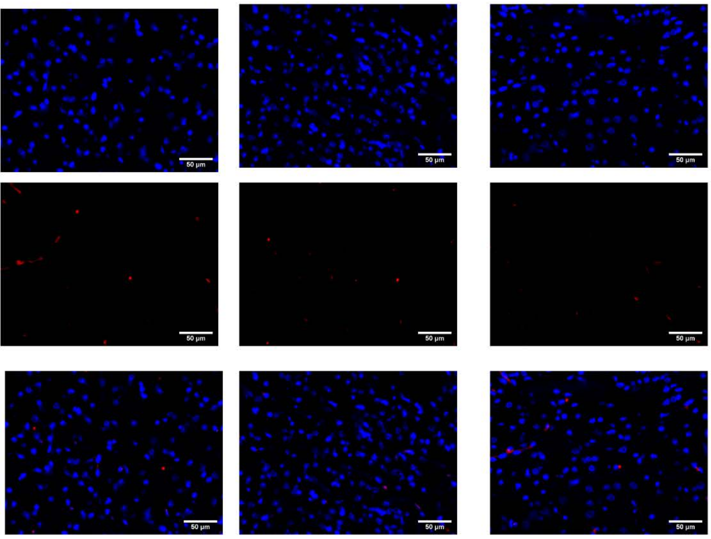

Cilostazol n=3

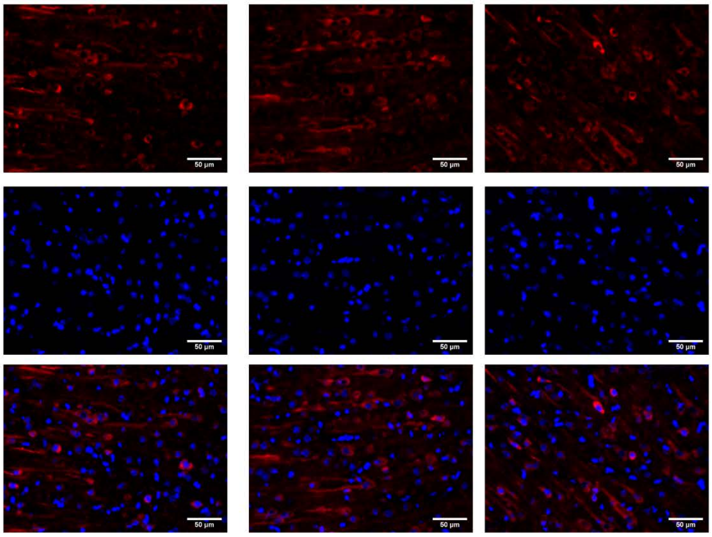

Sham n=3

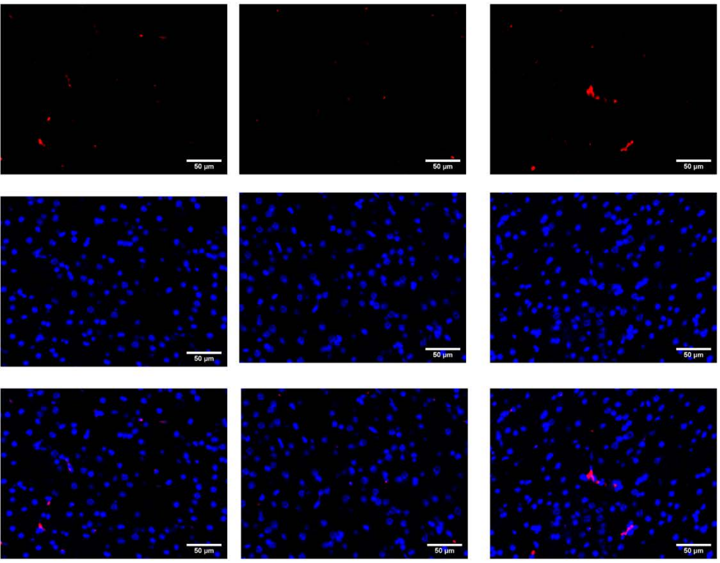

Aspirin n=3

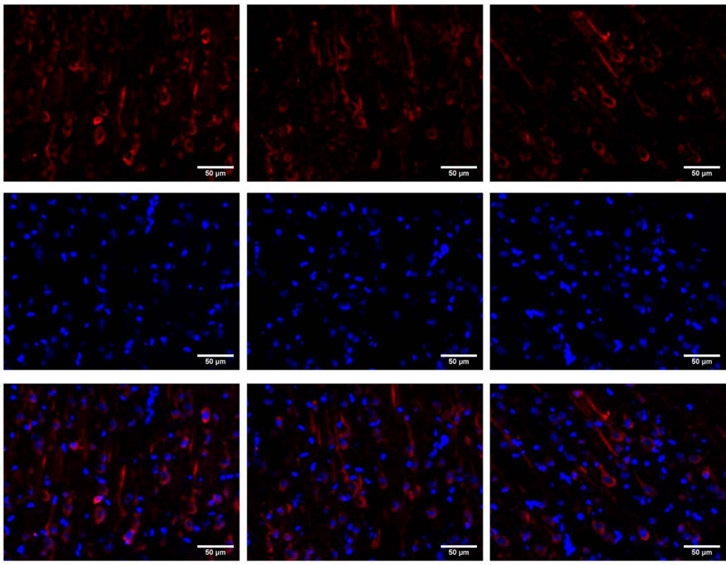

MCAO+CRF n=3

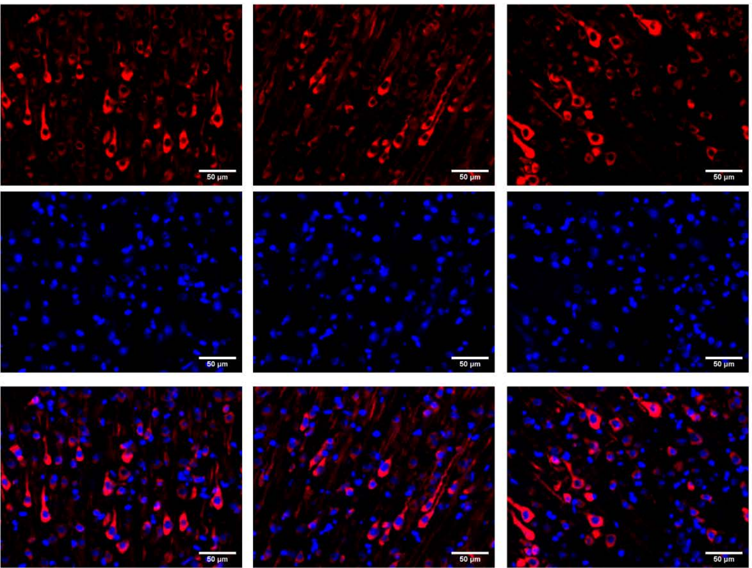

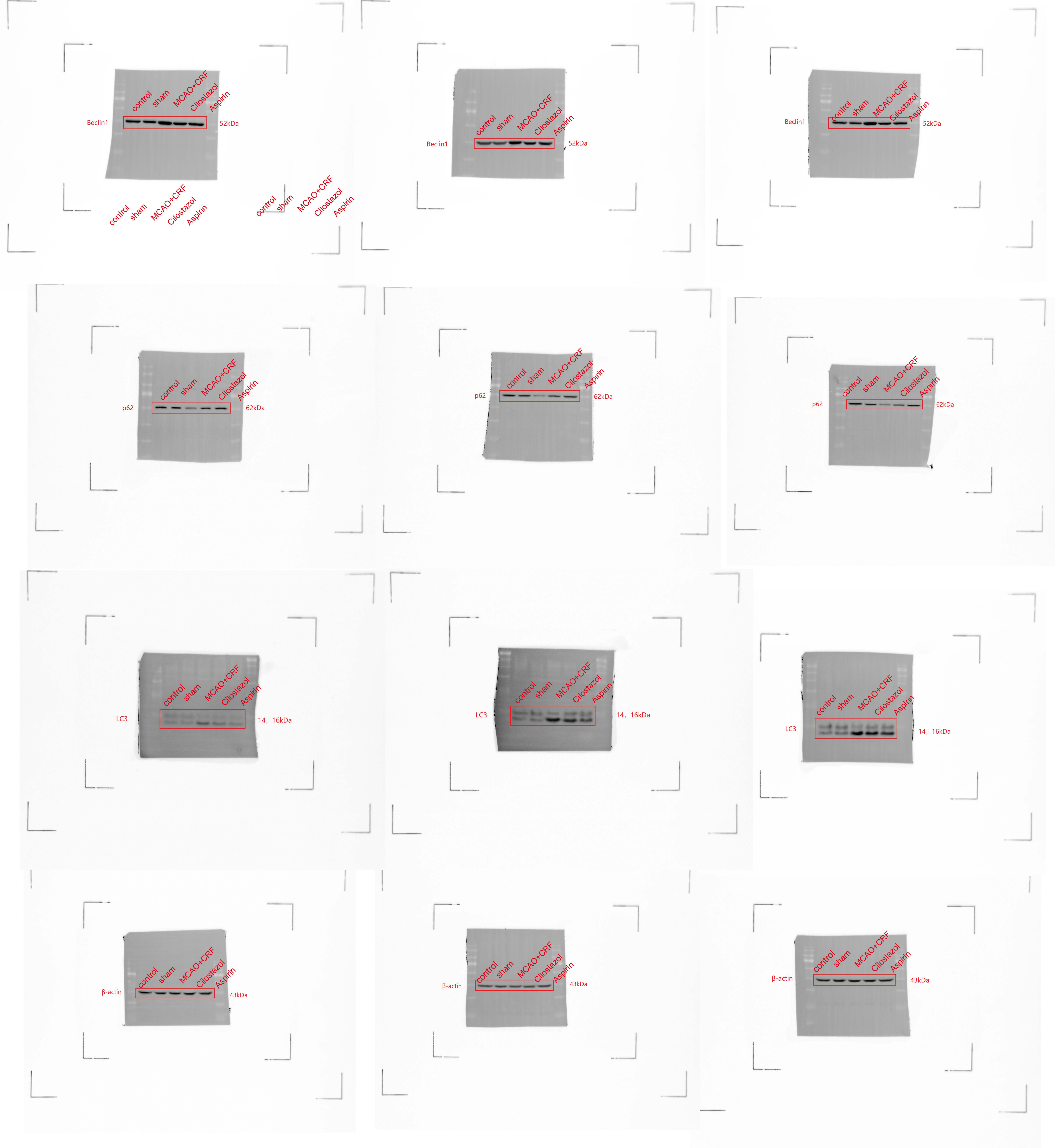

Beclin1
control
sham
MCAO+CRF
Cilostazol
Aspirin
52kDa
control
sham
MCAO+CRF
Cilostazol
Aspirin
control
sham
MCAO+CRF
Cilostazol
Aspirin
Beclin1
control
sham
MCAO+CRF
Cilostazol
Aspirin
52kDa
Beclin1
control
sham
MCAO+CRF
Cilostazol
Aspirin
52kDa
p62
control
sham
MCAO+CRF
Cilostazol
Aspirin
62kDa
p62
control
sham
MCAO+CRF
Cilostazol
Aspirin
62kDa
p62
control
sham
MCAO+CRF
Cilostazol
Aspirin
62kDa
LC3
control
sham
MCAO+CRF
Cilostazol
Aspirin
14，16kDa
LC3
control
sham
MCAO+CRF
Cilostazol
Aspirin
14，16kDa
LC3
control
sham
MCAO+CRF
Cilostazol
Aspirin
14，16kDa
β-actin
control
sham
MCAO+CRF
Cilostazol
Aspirin
43kDa
β-actin
control
sham
MCAO+CRF
Cilostazol
Aspirin
43kDa
β-actin
control
sham
MCAO+CRF
Cilostazol
Aspirin
43kDa

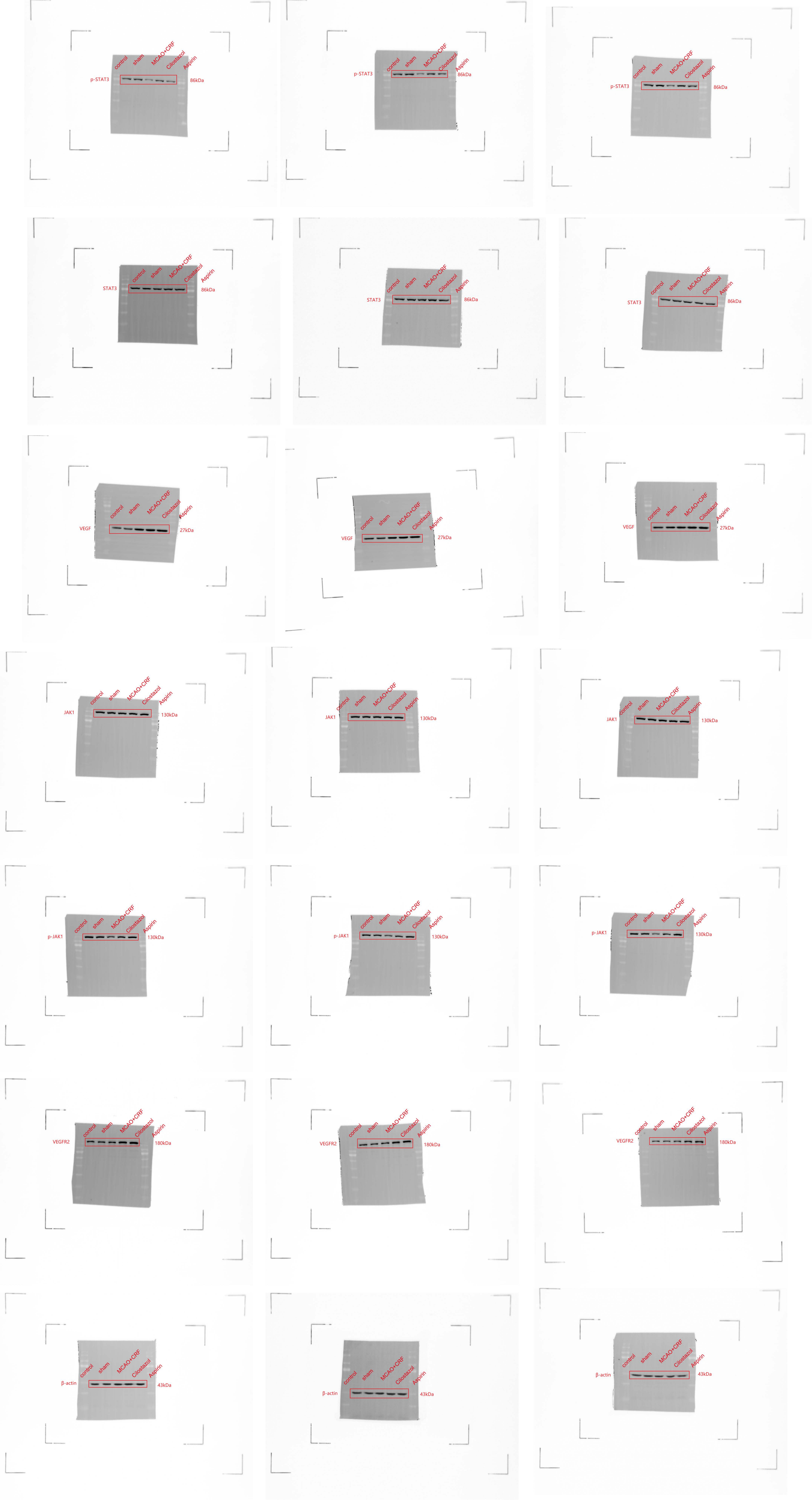
p-STAT3
control
sham
MCAO+CRF
Cilostazol
Aspirin
86kDa
p-STAT3
control
sham
MCAO+CRF
Cilostazol
Aspirin
86kDa
p-STAT3
control
sham
MCAO+CRF
Cilostazol
Aspirin
86kDa
STAT3
control
sham
MCAO+CRF
Cilostazol
Aspirin
86kDa
STAT3
control
sham
MCAO+CRF
Cilostazol
Aspirin
86kDa
STAT3
control
sham
MCAO+CRF
Cilostazol
Aspirin
86kDa
VEGF
control
sham
MCAO+CRF
Cilostazol
Aspirin
27kDa
VEGF
control
sham
MCAO+CRF
Cilostazol
Aspirin
27kDa
VEGF
control
sham
MCAO+CRF
Cilostazol
Aspirin
27kDa
JAK1
control
sham
MCAO+CRF
Cilostazol
Aspirin
130kDa
JAK1
control
sham
MCAO+CRF
Cilostazol
Aspirin
130kDa
JAK1
control
sham
MCAO+CRF
Cilostazol
Aspirin
130kDa
p-JAK1
control
sham
MCAO+CRF
Cilostazol
Aspirin
130kDa
p-JAK1
control
sham
MCAO+CRF
Cilostazol
Aspirin
130kDa
p-JAK1
control
sham
MCAO+CRF
Cilostazol
Aspirin
130kDa
VEGFR2
control
sham
MCAO+CRF
Cilostazol
Aspirin
180kDa
VEGFR2
control
sham
MCAO+CRF
Cilostazol
Aspirin
180kDa
VEGFR2
control
sham
MCAO+CRF
Cilostazol
Aspirin
180kDa
β-actin
control
sham
MCAO+CRF
Cilostazol
Aspirin
43kDa
β-actin
control
sham
MCAO+CRF
Cilostazol
Aspirin
43kDa
β-actin
control
sham
MCAO+CRF
Cilostazol
Aspirin
43kDa

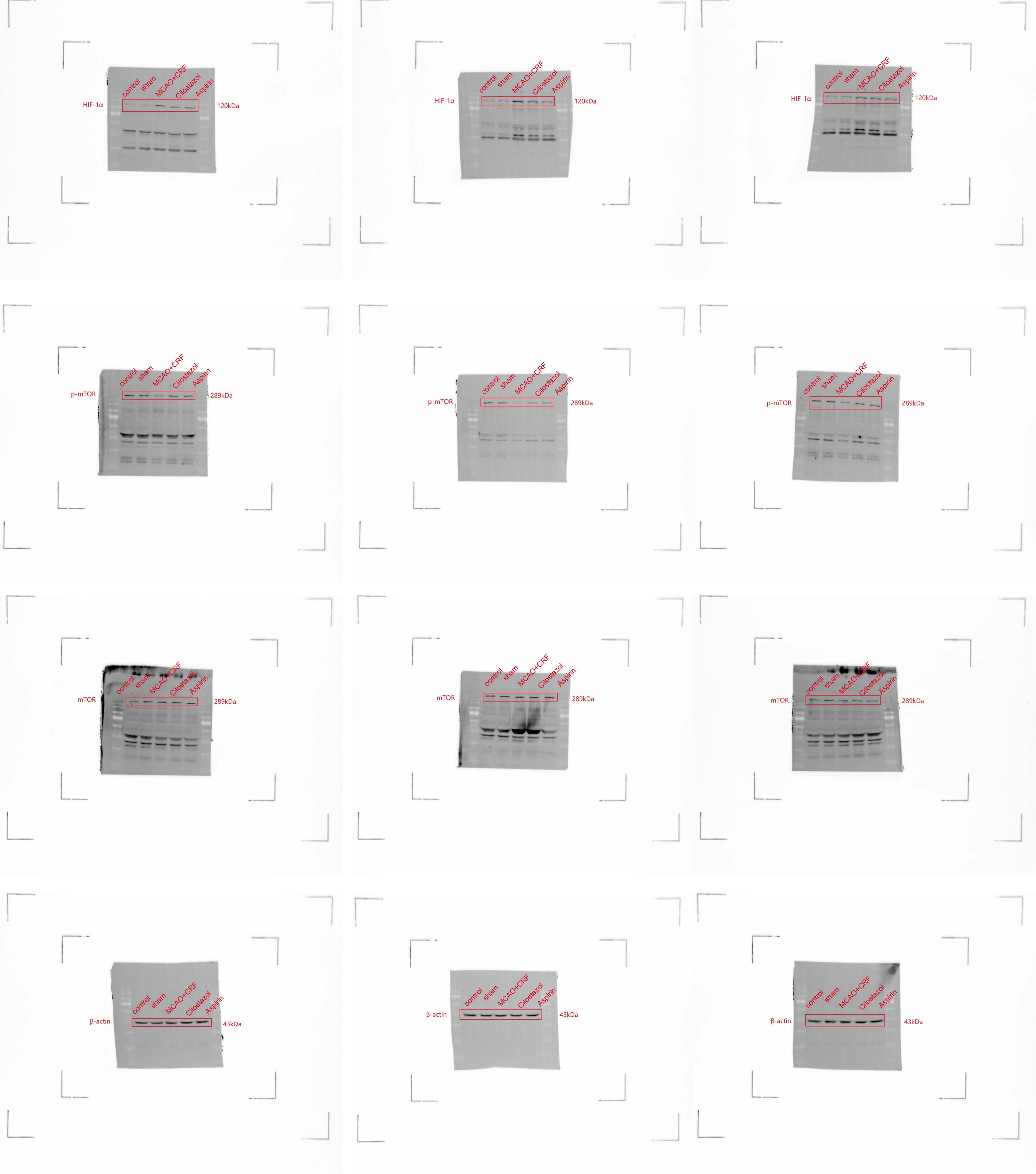
control
sham
MCAO+CRF
Cilostazol
Aspirin
HIF-1α
120kDa
control
sham
MCAO+CRF
Cilostazol
Aspirin
HIF-1α
120kDa
control
sham
MCAO+CRF
Cilostazol
Aspirin
HIF-1α
120kDa
control
sham
MCAO+CRF
Cilostazol
Aspirin
p-mTOR
289kDa
control
sham
MCAO+CRF
Cilostazol
Aspirin
p-mTOR
289kDa
control
sham
MCAO+CRF
Cilostazol
Aspirin
p-mTOR
289kDa
control
sham
MCAO+CRF
Cilostazol
Aspirin
mTOR
289kDa
control
sham
MCAO+CRF
Cilostazol
Aspirin
mTOR
289kDa
control
sham
MCAO+CRF
Cilostazol
Aspirin
mTOR
289kDa
control
sham
MCAO+CRF
Cilostazol
Aspirin
β-actin
43kDa
control
sham
MCAO+CRF
Cilostazol
Aspirin
β-actin
43kDa
control
sham
MCAO+CRF
Cilostazol
Aspirin
β-actin
43kDa
